# Gephyrin phosphorylation facilitates sexually dimorphic development and function of parvalbumin interneurons in the mouse hippocampus

**DOI:** 10.1038/s41380-024-02517-5

**Published:** 2024-03-19

**Authors:** Benjamin F. N. Campbell, Natalia Cruz-Ochoa, Kanako Otomo, David Lukacsovich, Pedro Espinosa, Andrin Abegg, Wenshu Luo, Camilla Bellone, Csaba Földy, Shiva K. Tyagarajan

**Affiliations:** 1 Institute of Pharmacology and Toxicology, University of Zürich, 8057 Zürich, Switzerland; 2 Laboratory of Neural Connectivity, Brain Research Institute, Faculties of Medicine and Science, University of Zürich, 8057 Zürich, Switzerland; 3 Adaptive Brain Circuits in Development and Learning (AdaBD), University Research Priority Program (URPP), University of Zürich, 8057 Zürich, Switzerland; 4 Department of Basic Neuroscience, University of Geneva, 1211 Geneva, Switzerland

**Keywords:** Autism spectrum disorders, Neuroscience

## Abstract

The precise function of specialized GABAergic interneuron subtypes is required to provide appropriate synaptic inhibition for regulating principal neuron excitability and synchronization within brain circuits. Of these, parvalbumin-type (PV neuron) dysfunction is a feature of several sex-biased psychiatric and brain disorders, although, the underlying developmental mechanisms are unclear. While the transcriptional action of sex hormones generates sexual dimorphism during brain development, whether kinase signaling contributes to sex differences in PV neuron function remains unexplored. In the hippocampus, we report that gephyrin, the main inhibitory post-synaptic scaffolding protein, is phosphorylated at serine S268 and S270 in a developmentally-dependent manner in both males and females. When examining *Gphn*^*S268A/S270A*^ mice in which site-specific phosphorylation is constitutively blocked, we found that sex differences in PV neuron density in the hippocampal CA1 present in WT mice were abolished, coincident with a female-specific increase in PV neuron-derived terminals and increased inhibitory input onto principal cells. Electrophysiological analysis of CA1 PV neurons indicated that gephyrin phosphorylation is required for sexually dimorphic function. Moreover, while male and female WT mice showed no difference in hippocampus-dependent memory tasks, *Gphn*^*S268A/S270A*^ mice exhibited sex- and task-specific deficits, indicating that gephyrin phosphorylation is differentially required by males and females for convergent cognitive function. In fate mapping experiments, we uncovered that gephyrin phosphorylation at S268 and S270 establishes sex differences in putative PV neuron density during early postnatal development. Furthermore, patch-sequencing of putative PV neurons at postnatal day 4 revealed that gephyrin phosphorylation contributes to sex differences in the transcriptomic profile of developing interneurons. Therefore, these early shifts in male-female interneuron development may drive adult sex differences in PV neuron function and connectivity. Our results identify gephyrin phosphorylation as a new substrate organizing PV neuron development at the anatomical, functional, and transcriptional levels in a sex-dependent manner, thus implicating kinase signaling disruption as a new mechanism contributing to the sex-dependent etiology of brain disorders.

## Introduction

Many psychiatric and developmental brain disorders present with disrupted excitation/inhibition (E/I) balance that can derive from dysfunction of inhibitory GABAergic neurotransmission [1–3]. Importantly, these diseases often display sex biases in prevalence and phenotypic presentation. For example, autism spectrum disorders (ASDs) and schizophrenia are more frequent in males [4], and present differently in males and females [5]. The mechanisms underlying sex differences in cortical and hippocampal function are unclear, due to a historical lack of inclusion of females in neuroscience research [5], and a focus on brain sex differences within sub-thalamic areas [6]. Neocortical circuit activity and plasticity are regulated by inhibitory GABAergic interneurons [7, 8], and disruption of GABAergic function is characteristic of brain disorders in both humans and animal models [9]. In particular, parvalbumin-type (PV) neurons, which mediate potent feedforward and feedback inhibition to control circuit function [7, 10], show disrupted PV protein expression and cell density in people with schizophrenia and ASDs [11–14]. Moreover, PV neurons may act as signaling hubs integrating environmental influences on disease phenotypes [15, 16]. PV neurons undergo prescribed developmental trajectories during prenatal and early postnatal stages, where precursors migrate tangentially and then radially from the medial ganglionic eminence (MGE) to cortical and hippocampal areas by birth, before initiating synapse formation and network integration to control adult neuron number and connectivity [7, 17, 18]. This sensitive period for PV neuron development is likely an important window for genetic and environmental information to influence sex-dependent signaling during brain development [19]. However, it is unknown whether PV neuron developmental trajectories are shared between males and females.

Candidate gene approaches have been employed to determine the impact of genetic risk variants on PV neurons using whole organism or cell-type-specific genetic knockout models [9]. However, gene product regulation is far more complex than simple transcriptional control, utilizing mRNA splicing, translational control, and post-translational modifications such as phosphorylation to impact protein function. Protein phosphorylation cascades transduce environmental signals to control neuron development, synaptic transmission, and plasticity [20]. Consequently, disruption of kinase activity including MAPK (ERK1/2), GSK3β, and mTOR signaling cascades contributes to certain schizophrenia and ASD phenotypes [21–24]. PV neurons in animal models of disease show disrupted mTOR and MAPK-driven phosphorylation [25–27], and manipulation of kinase activity in PV neurons influences neuronal survival and integration within local circuits [26–28]. Although, the downstream phosphorylation targets mediating these effects are unknown. Neuronal activity and genetic factors control the postnatal development and connectivity of PV neurons. In particular, interneuron excitability promotes their circuit integration and survival during postnatal apoptotic waves [29–33]. This interneuron cell death [7, 18] coincides with a shift in GABAergic transmission from excitatory to inhibitory during postnatal development [30, 34]. Therefore, GABAergic signaling during this sensitive period may affect PV development and consequent regulation of adult circuit function.

Gephyrin is the major inhibitory post-synaptic scaffolding protein at GABAergic and glycinergic synapses, regulating post-synaptic receptor organization [35]. Gephyrin acts by tethering inhibitory receptors to an apparatus composed of itself, collybistin, neuroligin 2, and additional proteins to organize and relay plasticity signals controlling inhibitory post-synaptic function [36, 37]. Gephyrin cluster formation and its ability to regulate synaptic strength are tightly coupled to its phosphorylation status at several key serine residues. Phosphorylation of gephyrin at serine 268 (S268) by ERK1/2, or serine 270 (S270) by GSK3ß, reduces gephyrin cluster size and density, decreasing GABAergic post-synaptic currents [38] by altering the dwell time of GABA_A_ receptors at mature synapses [39]. While gephyrin exonic microdeletions and missense mutations are associated with autism, schizophrenia, and epilepsy in humans [40–42], whether gephyrin phosphorylation is relevant for the establishment of inhibition during postnatal sensitive periods has not been examined.

In this study, we describe that gephyrin phosphorylation at S268 and S270 is regulated during postnatal development in the mouse hippocampus. We find that while WT males and females show differences in hippocampal PV neuron density, morphology, and function, blocking gephyrin phosphorylation using a constitutive phospho-null mouse model normalizes these sex differences from early postnatal development. Furthermore, we show using patch-sequencing of developing interneurons that blocking gephyrin phosphorylation alters interneuron electrophysiological function and eliminates sex differences in transcriptional profiles, implicating gephyrin phosphorylation as a putative mechanism for generating sexual dimorphism in inhibitory networks.

## Materials and methods

### Animals

Animal husbandry and animal experiments were performed in accordance with guidelines set by the Veterinary Office of the Canton of Zürich. Mice were housed under a 12 h:12 h light-dark cycle and given *ad libitum* access to food and water. None of the mouse strains used showed any signs of constraint under housing or experimental conditions. The Nkx2.1-Cre mouse line (C57BL/6J-Tg(Nkx2-1-cre)2Sand/J, Jackson ID 008661) drives Cre recombinase expression under the Nkx2.1 promoter in neuronal precursor cells of the medial ganglionic eminence (MGE) including somatostatin (SST) and parvalbumin (PV) interneuron subtypes [43]. The PV-Cre mouse line (B6;129P2-Pvalbtm1(cre)Arbr/J, Jackson ID 008069) drives Cre recombinase expression under the endogenous parvalbumin promoter [44]. The Ai14-tdTomato mouse line (B6.Cg-Gt(ROSA)26Sortm14(CAG-tdTomato)Hze/J, Jacskon ID 007914) includes a loxP-flanked stop cassette in front of CAG promoter-driven tdTomato gene in the Gt(ROSA)26Sor locus and drives tdTomato expression upon Cre-mediated recombination [45]. The *Gphn*^*S268/270A*^ mutant mouse was generated commercially as described below, other mouse strains were acquired from The Jackson Laboratory (Charles River Germany) and were genotyped according to available Jackson protocols. Confirmation of sex was performed using PCR-based methods [46]. The estrous state of adult female mice was determined by observing vaginal opening morphology and vaginal swab cytology [47].

### Generation of *Gphn*^*S268A/S270A*^ mutant mice

A mutant mouse line (*Gphn*^*S268A/S270A*^ mice) was generated where serine residues S268 and S270 were mutated to alanine residues at the endogenous genetic locus using a CRISPR-Cas9 approach (performed by Applied StemCell). Guide RNA targeting exon 8 of the genomic region corresponding to the principal isoform of gephyrin along with a single-stranded oligodeoxynucleotide (ssODN) and Cas9 mRNA was injected into the cytoplasm of C57BL/6 embryos, which were then implanted into CD1 surrogate mice. The ssODN template was designed to mutate S268 and S270 to alanine residues. Pups were sequenced and mice carrying the correct mutations (S268A TCA to GCT and S270A TCG to GCT) were crossed with wild-type (WT) mice to generate a heterozygous F1 generation carrying the mutated allele. The F1 generation was backcrossed into C57BL6/J mice for at least 8 generations before experimental cohorts were derived. To genotype mice, genomic DNA was amplified using the forward primer TCCATAAAGGTCAGCTAGAAGCGAAGAG and reverse primer AGCAGGTAAGTACGCTAGGCTGGAG, generating an 881 bp fragment in both wild-type, heterozygous, and homozygous mutant mice. Digestion with the restriction enzyme MwoI (ThermoFisher, ER1731) yields two fragments of 440 and 441 bp in the mutant allele only. Both homozygous and heterozygous *Gphn*^*S268A/S270A*^ mice are fertile and show no overt gross anatomical differences or constraints as determined from routine monitoring (e.g. normal weight, lifespan, health span, home cage behavior *et cetera*).

### Adult slice electrophysiology

#### Adult hippocampal pyramidal neuron recordings

Mice were sacrificed and 250 μm coronal slices were cut in solution containing (in mM): 91 choline chloride, 25 glucose, 25 NaHCO_3_, 7 MgCl_2_, 12 ascorbic acid, 3 sodium pyruvate, 2 KCl, 1.25 NaH_2_PO_4_, and 0.50 CaCl_2_, using a vibratome (Leica VT 1200 S, Germany). Slices were incubated for 20–30 min at 30° in oxygenated artificial cerebrospinal fluid (ACSF) containing (in mM): 119 NaCl, 2.5 KCl, 1.3 MgCl_2_, 2.5 CaCl_2_, 1.0 NaH_2_PO_4_, 26 NaHCO_3_, and 11 glucose, then subsequently kept at room temperature (RT). Recordings were made under an upright microscope (Olympus BX51WIF, Tokyo, Japan) with infrared differential interference contrast (DIA) optics. Whole-cell recordings were obtained from pyramidal neurons in CA1 at RT using borosilicate patch pipettes (Harvard apparatus, 30–0068) pulled to a resistance of 3–4 MΩ filled with internal solution containing (in mM): 130 CsCl, 4 NaCl, 2 MgCl_2_, 1.1 EGTA, 5 HEPES, 2 Na_2_ATP, 5 sodium creatine phosphate, 0.6 Na_3_GTP, and 0.1 spermine. To isolate mIPSCs, 1 μM tetrodotoxin, NBQX (10 mM), and APV (50 mM) were added to the bath solution. Data were collected using a Multiclamp700B amplifier and Clampex software (Axon Instruments), filtered at 2.2 kHz, and digitized at 10 Hz. Miniature EPSCs were analyzed using Igor Pro software 6.3 and 8.0 (Wavemetrics). Recordings were discarded if series resistance was above 20 MΩ or changed by >20%.

#### Adult hippocampal parvalbumin neuron recordings

Adult (10–12 weeks) male and female WT and *Gphn*^*S268A/S270A*^ mice crossed into PV-Cre: Ai14-tdT lines were used to visualize CA1 pyramidal layer PV neurons. Mice were anesthetized under isoflurane and transcardially perfused with ice-cold oxygenated ACSF containing (in mM): 126 NaCl, 2.5 KCl, 10 glucose, 1.25 NaH_2_PO_4_, 2 MgCl_2_, 2 CaCl_2_ and 26 NaHCO_3_. Brains were quickly harvested and 300 μm coronal slices were prepared using a vibrating microtome (Microm HM 650 V; Thermo Scientific) in oxygenated ice-cold ACSF containing (in mM): 85 NaCl, 75 sucrose, 2.5 KCl, 25 glucose, 1.25 NaH_2_PO_4_, 4 MgCl_2_, 0.5 CaCl_2_ and 24 NaHCO_3_. Slices were then incubated for 1 h at 34 °C and subsequently maintained in the same solution at RT with continuous oxygenation. During recording, slices were placed under an upright microscope (Zeiss Axio Examiner) equipped with DIA optics. A tdTomato filter setting was used to identify PV neurons. Whole-cell recordings were obtained from PV neurons in CA1 at RT using borosilicate patch pipettes (30-0053; Harvard Apparatus) with a resistance of 4–7 MΩ, prepared with a vertical puller (PC-100; Narishige), filled with internal solution containing (in mM): 95 K‐gluconate, 50 KCl, 10 HEPES, 4 Mg‐ATP and 0.5 Na‐GTP 10 phosphocreatine, at pH 7.2. To evaluate intrinsic excitability, a 1.5 s depolarizing current of increasing amplitude was injected in −50 pA steps. Data were acquired using HEKA EPC10 amplifier and PatchMaster software (HEKA Elektronik) at a sampling frequency of 50 kHz. Recordings were discarded if series resistance was above 25 MΩ or changed by >20%. Data extraction and analysis were performed using custom Python and MATLAB scripts (MathWorks).

### Tissue collection for lysate preparation

Animals were euthanized by cervical dislocation and removal of the head. Tissues were acutely dissected on ice and flash-frozen in liquid nitrogen before freezing at −80 °C. Tissues were weighed and homogenized in 20× weight/weight lysis buffer containing protease (cOmplete Mini, Roche) and phosphatase inhibitor (Phosphatase inhibitor cocktails 2 and 3, Sigma) cocktails. For immunoblotting experiments, RIPA lysis buffer was used (150 mM NaCl, 0.1% SDS, 1% NP-40, 1% deoxycholate, 50 mM Trizma pH 7.5); for immunoprecipitation experiments, EBC buffer was used (50 mM Trizma base, 120 mM NaCl, 0.5% NP-40). Sample protein concentration was determined using a BCA assay kit (Pierce) to ensure equal loading volume of protein. Samples were prepared for immunoblotting in 5x Laemmli buffer (50 mM Trizma pH 6.8, 0.1 M DTT, 0.2% bromophenol blue, 10% glycerol) by heating at 95 °C for 5 min.

### Immunoblotting

An equal concentration of protein was loaded for all samples within an experiment as determined by BCA assay (Pierce BCA Assay). SDS-PAGE was performed using an 8–10% polyacrylamide gel in Tris-glycine running buffer and transferred onto PVDF membranes (Amersham Hybond) using a wet transfer apparatus (BioRad) in a 20% methanol-sodium phosphate buffer containing SDS. Membranes were blocked in 5% blocking solution (Roche) in TBST (50 mM Tris, 150 mM NaCl, 1% Tween, pH 7.5) before overnight incubation in the primary antibody at 4 °C in 5% blocking solution in TBST. For most experiments, fluorescent secondary antibodies were used to detect signal using an Odyssey CLx system (LI-COR Biosciences). For the detection of phospho-gephyrin, HRP-conjugated secondary antibodies were used along with a luminol-based detection kit (Supersignal Femto West, Thermofisher) and a CCD-based imaging system (FujiFilm). Quantification of raw intensity signal was performed in FIJI (ImageJ). The phosphorylation of gephyrin at S268 and S270 is always represented relative to the total gephyrin protein for analysis. For representative blot images, intensity signals were inverted and represented as dark signals against a light background. All antibodies (Table 1) have been previously validated for their specificity.

**Table 1:** List of primary antibodies and concentrations used for immunoblotting.

Antibody	Species	Clone	Dilution	Company	Identifier	RRID
Anti-actin	Rabbit	Polyclonal	1:6000	Sigma	A2066	AB_476693
Anti-gephyrin	Mouse	3B11	1:1000	Synaptic Systems	147111	AB_887719
Anti-gephyrin p268	Rabbit	Polyclonal	1:500	Homemade	[38]	ND
Anti-gephyrin p270	Rabbit	Polyclonal	1:500	Homemade	[38]	ND

Dilution values correspond to manufacturer-recommended reconstitution concentrations. Research Resource identifiers are indicated where extant.

*ND* no data.

### Tissue immunofluorescence

Animals were anaesthetized with intraperitoneal injections of pentobarbital before transcardial perfusion with oxygenated, ice-cold artificial cerebrospinal fluid containing (in mM) 125 NaCl, 2.5 KCl, 1.25 NaH_2_PO_4_, 26 NaHCO_3_, 25 D-glucose, 2.5 CaCl_2_, and 2 MgCl_2_). Perfused brains were dissected and post-fixed in 150 mM phosphate-buffered saline (PBS) containing 4% paraformaldehyde (PFA, pH 7.4) for 90 min at 4 °C. Tissue was cryoprotected overnight in PBS containing 30% sucrose at 4 °C, then cut into 40 µm thick sections using a sliding microtome. Sections were stored at −20 °C in an antifreeze solution (50 mM sodium phosphate buffer with 15% glucose, 30% ethylene glycol at pH 7.4) until use. Early-postnatal tissue was not perfused, but post-fixed for 24 h before sucrose cryoprotection and cut at 50 µm. For immunofluorescence experiments, sections were washed 3 × 10 min under gentle agitation in TBST (50 mM Tris, 150 mM NaCl, 1% Tween, pH 7.5) before overnight incubation in primary antibody (Table 2) solution (TBST containing 0.2% Triton X-100 and 2% NGS). Sections were then washed 3 × 10 min and incubated for 30 min at RT with secondary antibodies in TBST solution with 2% NGS. Sections were washed again 3 × 10 min in TBST before transfer to PBS and mounting onto gelatine-coated slides using DAKO mounting medium. All tissue morphological analysis, image acquisition, processing, and analysis were executed blind to condition.

**Table 2:** List of primary antibodies and concentrations used for immunofluorescence.

Antibody	Species	Clone	Dilution	Company	Identifier	RRID
Anti-synaptotagmin 2	Rabbit	Polyclonal	1:2000	Synaptic Systems	105123	AB_2199465
Anti-parvalbumin	Guinea pig	Polyclonal	1:1000	Immunostar	24428	AB_572259
Anti-somatostatin	Rabbit	H-106	1:4000	Santa Cruz	sc-13099	AB_2195930
Anti-GABRG2	Guinea pig	Polyclonal	1:2000	Homemade	NA	ND

Dilution values correspond to manufacturer-recommended reconstitution concentrations. Research Resource identifiers are indicated where extant.

*ND* no data.

### Cell counting

Images used for cell number quantification experiments were acquired on a Zeiss LSM 700 laser scanning confocal microscope operating Zen image acquisition software (Zen 2011) using either 10× or 40× immersion objectives. Identical imaging settings were used when comparing groups in a given experiment. ROIs encompassing different anatomical layers were defined based on DAPI staining. Single and double positive cells for different fluorescent markers were counted and compiled manually. Only cells whose soma lay within the confocal Z stack were considered for counting. For adult cell counting experiments, 10–12-week-old male and female WT and *Gphn*^*S268A/S270A*^ mice crossed into PV-Cre: Ai14-tdT lines were used to visualize CA1 pyramidal layer PV neurons. For early postnatal cell counting experiments at p0, p4, and p7, male and female WT and *Gphn*^*S268A/S270A*^ mice crossed into Nkx2.1-Cre: Ai14-tdTomato lines were used for visualization of MGE-derived interneurons. For experiments measuring parvalbumin protein fluorescent intensity, tdTomato fluorescence was used to demarcate the soma to generate ROIs. Consecutive sections were imaged, and averages were compared between individuals.

### Synaptic analysis

Images used for synapse quantification experiments were acquired on a Zeiss LSM 800 laser scanning confocal microscope operating Zen image acquisition software (Zen 2011) using a 40× oil immersion objective. Identical imaging settings were used when comparing groups in a given experiment. Synaptic quantification and colocalization analysis were performed using a previously described custom ImageJ macro [48]. Depending on the figure, 6–12 consecutive sections were imaged and averages were compared between individuals.

### Behavioral testing

Mice were gently handled daily for at least 5–7 days before the experiment. Mice were brought to the experimental room 30 min prior to experiment onset. Smell cues were removed with 70% ethanol between experiments. All behavioral testing was performed under dim illumination during the light phase. Experimentation, recording, and analysis was performed blind to genotype.

#### Open field test

A 40 × 40 × 25 cm arena was constructed out of white plastic, and a camera was positioned 60 cm above the base. Mice were recorded for 20 min using a digital camera (GoPro) and mouse motion was analyzed using Noldus software. Mouse position, speed, total movement, and the time spent in the center (20 × 20 cm) were recorded. 3 male WT and one female *Gphn*^*S268A/S270A*^ mouse were excluded for attempting to jump out of the arena.

#### Elevated plus maze (EPM) test

The arena was a standard half-opaque polycarbonate maze positioned 0.5 m off the floor, with an observing digital camera 80 cm above the top of the maze, recording at a frame rate of 30 fps. The arena was divided into two covered and two non-covered arms as well as a center uncovered section. The number of entries and time spent in each area was determined manually using Kinovea video playback software (Kinovea v. 0.8.15).

#### Novel object recognition (NOR) test

The arena consisted of a 31.5 × 31.5 cm area with 23.5 cm high walls (opaque white laminate). Mice were recorded using a digital camera suspended 45 cm above the top of the arena acquiring at 24 fps. A piece of thick black tape was placed along one wall to provide orientation to the mice. Objects (red-capped flask with blue filling and shiny tape, or blue-capped bottle with green tape) were chosen for their differences in shape and color and were placed in different corners away from the wall. Four identical copies of each object were created and wiped of odorants between uses. Mice were acclimatized to the arena for 5 min without objects twice before testing. During the first day of testing (object exposure), mice were allowed 10 min to explore the objects. Mice were returned to the arena 24 h later after one of the objects was replaced with a novel object. Behavior was analyzed manually using Kinovea, where sniffing and touching of the objects within 1 cm was recorded as exploration while climbing on the objects or standing beside was not. Mice that did not achieve 20 s of total exploration time (criterion time) during exploration and testing phases were excluded *a prior*i from analysis. For males, all mice reached criterion (*n* = 5 WT, 7 *Gphn*^*S268A/S270A*^); for females, 4 mice failed to reach criterion during the test session (*n* = 2 WT, *n* = 2 *Gphn*^*S268A/S270A*^). Video was acquired at 24 fps and the first 5 min of exposure to the object were analyzed. The time to reach criterion and time spent exploring each object was analyzed. The discrimination index (DI) indicates relative preference for novel or non-novel objects: ${DI}=\frac{({Novel\; object\; exploration\; time}-{Familiar\; object\; exploratoin\; time})}{{Total\; exploration\; time}}$.

#### Object location test

A 40 × 40 × 25 cm arena was constructed out of while plastic and a camera positioned 60 cm above the base. After 5 days of acclimatization to the arena for 5 min per day, mice were allowed to explore two objects placed 5 cm from two corners and allowed 10 min of exploration. Mice were re-exposed to the objects the following day after one of the objects was moved to the opposite corner (randomized between mice). Spatial cues were created using patterns of red and green tape. The first 5 min of exploration were analyzed manually using Kinovea playback software. Video was acquired at 24 fps. One *Gphn*^*S268A/S270A*^ female, two WT female one WT male, and one *Gphn*^*S268A/S270A*^ male were excluded from the final analysis for failing to reach the exploration criterion time. Exploration and discrimination index were measured as in the NOR test.

#### Contextual fear conditioning

Prior to conditioning, animals were transported to a holding area adjacent to the experimental room and handled individually for two minutes per day for five consecutive days. On the conditioning day, animals were brought to the holding area one hour before conditioning. The animals were then transferred to a rectangular 30 × 25 × 25 cm (W, D, H) fear conditioning chamber with a conductive metal grid floor (Med Associates) representing “Context A”. 0.7 mA electric shocks lasting two seconds each were administered 120, 180, and 240 s after the animals were placed in the chamber. Animals were removed from the chamber 60 s after the final shock and placed back in their home cage. One hour after conditioning, the animals were transported back to their homeroom. Memory specificity was tested two days later by placing the animals back in the conditioning chamber in which the walls had been replaced by a triangular striped insert and the grid floor covered by a white un-textured plate for a total of five minutes (“Context B”). One day following the memory specificity test, the overall strength of the contextual memory was tested by placing the animal in the original conditioning chamber for five minutes (“Context A”). Videos were recorded from a top-down perspective and freezing was quantified using the ezTrack software [49] with a minimum bout duration of one second. Freezing was calculated as a percentage of total test time. For each animal, a discrimination index was calculated from the freezing values ${DI}=\frac{(A-B)}{{Max}(A,\,{B})}$ .

### Patch-sequencing of putative CA1 PV neurons at p4

#### P4 slice electrophysiology

Male and female WT and *Gphn*^*S268A/S270A*^ mice crossed into Nkx2.1-Cre: Ai14-tdTomato mouse lines for visualization of MGE-derived interneurons were sacrificed by 10:00 am on p4, and 300 μm coronal brain slices of the dorsal hippocampus were prepared using a Leica vibratome in oxygenated (95% O_2_, 5% CO_2_) ice-cold sucrose-containing ACSF containing the following (in mM): 85 NaCl, 75 sucrose, 2.5 KCl, 25 glucose, 1.25 NaH_2_PO_4_, 4 MgCl_2_, 0.5 CaCl_2_ and 24 NaHCO_3_. Slices were incubated for 1 h at 34 °C and subsequently held at RT in the same solution until recording. Slices were visualized using an upright microscope (Olympus, BX-61WI) with infrared DIC optics using a camera (ORCA‐Flash 4.0 CMOS, Hamamatsu). Electrophysiological recordings were made at 37 °C in oxygenated ACSF containing (in mM): 126 NaCl, 2.5 KCl, 10 glucose, 1.25 NaH_2_PO_4_, 2 MgCl_2_, 2 CaCl_2,_ and 26 NaHCO_3_). Whole-cell recordings were obtained from tdT+ interneurons in the CA1 pyramidal cell layer using patch pipettes (GC150F‐10; Harvard Apparatus) created with a puller (PC-100; Narishige) with a resistance of 5 MΩ, filled with intracellular solution containing (in mM): 95 K‐gluconate, 50 KCl, 10 HEPES, 4 Mg‐ATP and 0.5 Na‐GTP 10 phosphocreatine, at pH 7.2. Cells were held at −45 mV for voltage-clamp recordings and at −60 mV for current-clamp recordings. To evaluate intrinsic excitability, a 1.5 s depolarizing current of increasing amplitude was injected. Data were obtained using MultiClamp700B amplifiers (Molecular Devices, Sunnyvale, CA). Signals were filtered at 10 kHz with a Bessel filter and digitized (50 kHz) using a Digidata 1440 A and pCLAMP 10 (Molecular Devices). Recordings were discarded if the series resistance changed significantly or reached 20 MΩ. Data extraction and analysis were performed using custom Python scripts.

#### Voltage-clamp recordings

A 5 mV voltage step was applied to measure the resulting current amplitude over time. Input resistance was calculated as the applied voltage divided by the difference between the pre-voltage step steady state, and during-voltage step steady-state current. Series resistance was calculated as the applied voltage divided by the difference between the pre-voltage step steady-state current, and the lowest current value during the voltage step. Capacitance was calculated as the area between the voltage step steady-state current level, and the during-voltage current graph, divided by the voltage step.

#### Current-clamp recordings

We applied current injections in steps (traces) over time periods of 1.5 s and measured the spiking response from the cell. We calculated a LOWESS (smoothed) fit of the voltage versus time graph and called it the baseline voltage. We defined a spike as existing if the voltage level was at least 1.5 times higher than the noise (relative to the baseline) of the trace at the location. As a quality control step, we eliminated any traces where there was no current, or negative current injection and we saw two or more spiking signals, and traces where the pre-current injection step was too noisy (standard deviation of a LOWESS fit of the span >1.0). We calculated the AP amplitude and AP half-width on the first trace with at least 3 peaks, taken as the means of the amplitudes and halfwidths of the individual peaks. The AP attenuation was calculated on the first trace with at least 7 peaks, and taken as the ratio of the average amplitude of the first 3 and last 3 peaks. For each trace, we defined the AP firing frequency as the number of spikes during the current injection divided by the time span of the current injection.

#### RNA sample preparation and sequencing

RNA sample processing and sequencing was performed as described [50]. Briefly, neuronal cytoplasm was aspirated into a patch pipette and transferred into sample buffer. Clontech’s SMARTer Ultra Low RNA Input v4 kit was used to prepare single-cell mRNA, and library preparation used the Illumina Nextera XT DNA Sample Preparation kit. Pooled libraries were sequenced with NextSeq 300 high-output kit using an Illumina NextSeq 500 sequencer with 2 × 75 paired-end reads.

#### Processing of RNA-seq data

After sequencing, raw sequencing reads were aligned to the Ensembl GRCm38 reference transcriptome (Version 95), using Kallisto’s quant command with 10 bootstraps. For convenience, Ensembl stable IDs were converted to gene symbols using a reference file generated by biomaRt. In the few cases where different Ensembl gene IDs identified the same gene symbol, transcript per million (TPM) levels were summed.

#### Sequencing data quality control

All data analysis was performed using R and Python. Firstly, in each cell, the number of unique genes and the number of aligned reads were calculated. Secondly, the median and median absolute deviation of these two values across all cells was calculated. Cells with values more than three absolute deviations below the median were removed as failing quality control. After noticing potential microglia contamination in our data, we selected a subset of our data containing only known microglia markers. We then ran an embedding on this data (umap, nearest_neighbors = 15), and found 2 distinct clusters (both visually, and by k-means clustering). Highlighting our data by the expression levels of individual marker genes showed that the microglia markers were consistently expressed in the smaller cluster (*n* = 13), but not in the larger cluster (*n* = 98). Therefore, we labeled the cells in the smaller clusters as microglia-contaminated and removed them from further analysis.

#### Differential gene expression analysis

Transcripts-per-million (TPM) normalization of transcripts was calculated by a built-in Kallisto function. For calculating differentially expressed (DE) genes, we first read in Kallisto’s output using Tximport, to account for uncertainty in alignment. We then imported the results to edgeR and used a quasi-likelihood test on all genes that were expressed at a level of TPM > 15 in at least ten cells in the two groups being compared. Genes were labeled as DE if there was a fold difference of at least 2 (absolute value of log_2_ fold difference>1) in average expression, at a significance of *P*-adjusted <0.05. RNA sequencing data was deposited on the Gene Expression Omnibus repository https://www.ncbi.nlm.nih.gov/geo/query/acc.cgi?acc=GSE197607. Gene ontology analysis was performed using WebGestalt (https://www.webgestalt.org/), although no significant enrichment categories were found for differentially expressed transcripts. Functional gene product classification was assessed by searching the GeneCards database (https://www.genecards.org/).

### Statistical analysis and visualization

Data were organized using Microsoft Excel (Microsoft Office 2016). All statistical analysis and plotting were performed using Prism 8 (GraphPad). Statistical parameters, sample sizes, and tests are indicated in the figure legends or tables in which the data appears. Sample sizes were determined based on previous power calculations of cohort sizes for respective experiments in the lab. Parametric tests were used when the data was determined to be normal using the Shapiro-Wilks test for ANOVA, or when samples showed unequal variance. Tukey’s post-hoc test was used for One-way ANOVA multiple comparisons. For two-way ANOVA multiple comparisons, the Sidak method was used to test the difference between each group. Non-parametric tests were used when these requirements were not satisfied. Chi-Squared analyses were used when dependent and independent variables were categorical (e.g., Mendelian birth ratios). Figures were arranged using an open-source graphics editor Inkscape (1.0). All plotted data and related statistical tests are available in the supplementary file “Supplementary Tables”, with data for each figure/supplementary figure in separate tabs.

## Results

### Gephyrin phosphorylation at S268 and S270 is downregulated during hippocampal development

**Fig. 1:**
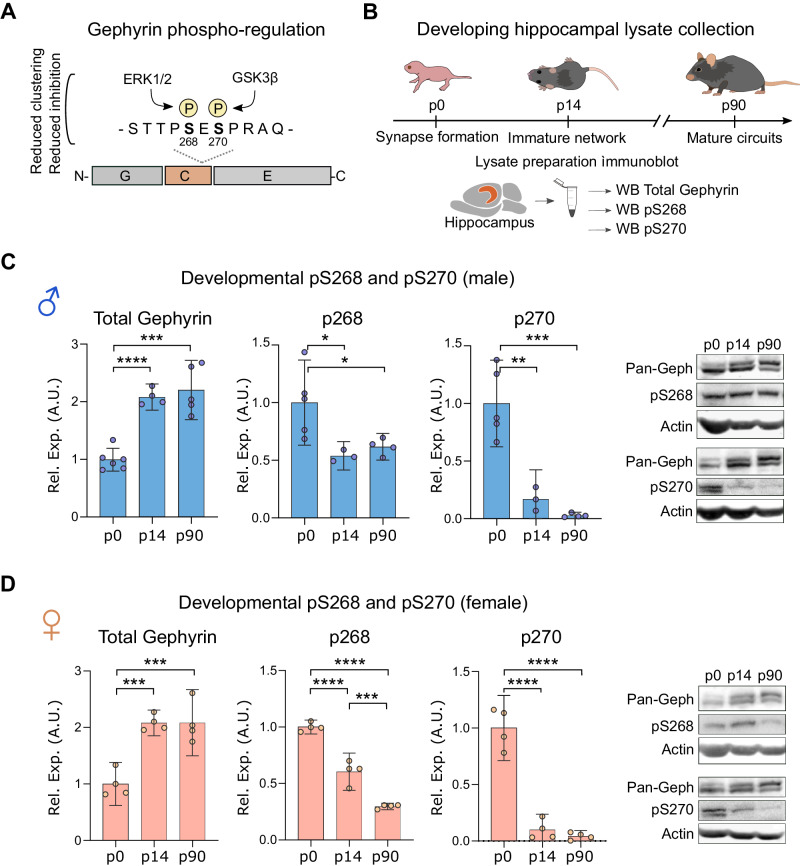
Gephyrin phosphorylation is developmentally regulated in the hippocampus of male and female mice. **A** Gephyrin is phosphorylated at S268 (ERK1/2) and at S270 (GSK3ß) to downregulate gephyrin clustering and inhibitory neurotransmission. **B** Timeline of hippocampal lysate collection at postnatal days 0, 14, and 90. **C**, **D** Hippocampal immunoblots for total gephyrin (*male: F* = *31.37, p* < *0.0001; female: F* = *22, p* = *0.0003*), gephyrin pS268 (*male: F* = *6.199, p* = *0.0203; female: F* = *118.6, p* < *0.0001*), and gephyrin pS270 (*male: F* = *28.4, p* < *0.001; female: F* = *84.15, p* < *0.0001*). Phosphorylated gephyrin is represented relative to total gephyrin. Statistics: *n* = 3–7 hippocampi from individual mice per group, for p0 tissue collection, hippocampi from two mice were pooled per one sample. One-way ANOVA, Tukey’s post-hoc test. *p < 0.05, **p < 0.01, ***p < 0.001, ****p < 0.0001. Bars, mean ± SD.

Gephyrin forms inhibitory post-synaptic scaffolds by self-association via its G- and E-domains, while post-transcriptional modifications of the C-domain alters gephyrin clustering. Phosphorylation of gephyrin C-domain S268 and S270 by the kinases ERK1/2 and GSK3β reduces GABAergic transmission [38, 51] (Fig. 1A). However, whether this phosphorylation is regulated during important stages of brain development has not been investigated. We assessed relative gephyrin phosphorylation by comparing immunoblotting of phospho-S268 or S270 gephyrin signal to total (pan-gephyrin) using specific antibodies [38]. We probed male and female hippocampal lysates from p0 (when inhibitory synapses start to form), at p14 (after major interneuron apoptosis), and at p90 (when inhibitory networks are mature) (Fig. 1B). We observed a stark downregulation of gephyrin phosphorylation at S268 and S270 during development in both males and females (Fig. 1C, D), with the strongest decrease occurring within the first two postnatal weeks. In adult mice, we detected greater S268 phosphorylation in male versus female hippocampi (Supplementary Fig. 1A). Neither S268 nor S270 phosphorylation showed estrous cycle-dependent regulation in females (Supplementary Fig. 1B). As gephyrin phosphorylation at S268 and S270 alters GABAergic signaling, these results suggest that downregulation of gephyrin phosphorylation between p0 and p14 may influence the development of inhibitory networks in the hippocampus.

### Gephyrin phosphorylation reduces hippocampal inhibition and is required for sexually dimorphic PV neuron density

**Fig. 2:**
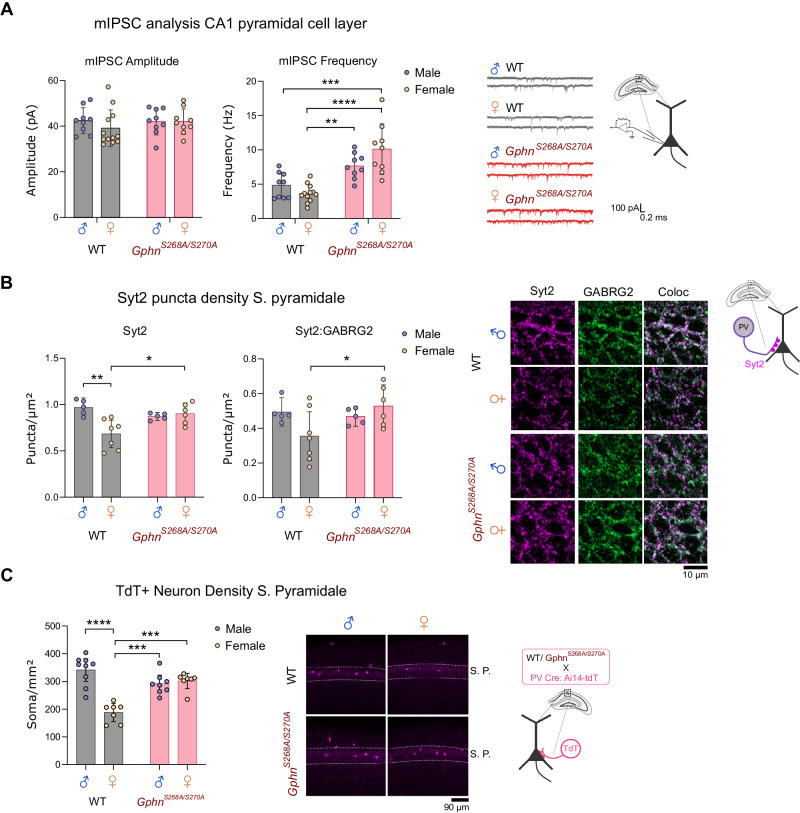
Altered hippocampal inhibition and disruption of sexually dimorphic PV neuron connectivity and density in *Gphn*^*S268A/S270A*^ mice. **A** Patch-clamp recording of CA1 pyramidal cells of acute hippocampal slices from WT and *Gphn*^*S268A/S270A*^ male and female mice: mIPSC amplitude *(Interaction: F(1,35)* = *0.6442, p* = *0.4276; Sex: F(1,35)* = *0.3402, p* = *0.5635; genotype: F(1,35)* = *0.6285, p* = *0.4332*) and mIPSC frequency (*Interaction: F(1,35)* = *6.129,p* = *0.0183; Sex: F(1,35)* = *39.92, p* < *0.0001; genotype: F(1,35)* = *0.7358, p* = *0.3968)* **B** Density of Syt2+ puncta in hippocampal CA1 pyramidal cell layer *(Interaction: F(1,19)* = *10.76, p* = *0.0039; genotype: F(1,19)* = *6.998, p* = *0.0160; sex: F(1,19)* = *1.593, p* = *0.2222)*, and colocalization with post-synaptic GABA_A_ receptor subunit γ2 (GABRG2) *(Interaction: F(1,19)* = *4.58,p* = *0.0462; genotype: F(1,19)* = *0.7299, p* = *0.4036; sex: F(1,19)* = *2.509, p* = *0.1297)*. **C** Density of tdTomato (tdT+) PV neurons in the stratum pyramidale (S.P.) of the hippocampal CA1 *(Interaction: F(1,27)* = *30.67,p* < *0.0001; genotype: F(1,27)* = *23.22, p* < *0.0001; sex: F(1,27)* = *4.686, p* = *0.0394)*. Statistics: Panel **A** *n* = 9–12 individual cells recorded across 2–4 mice per group. Panels **B**, **C**: *n* = 7–12 individual mouse average values (6–10 sections per mouse). All panels: two-way ANOVA with Sidak’s post-hoc test comparing all groups. *p < 0.05, **p < 0.01, ***p < 0.001, ****p < 0.0001. Bars, mean ± SD.

Previous studies indicate that preventing gephyrin phosphorylation at S268 and S270 increases the frequency and amplitude of miniature inhibitory post-synaptic currents (mIPSCs) in hippocampal neurons and organotypic slices [52], although, the functional effect of blocking gephyrin phosphorylation throughout development is unknown. The *Gphn*^*S268A/S270A*^ phospho-null mouse is a useful model to study the effect of preventing gephyrin phosphorylation at these sites (see “Methods” section), as they carry site-specific serine to alanine mutations at S268 and S270 at the endogenous genetic locus (Supplementary Fig. 2A). These mice are born at Mendelian sex and genotype ratios (Supplementary Fig. 2B) and present normal brain and body weights (Supplementary Fig. 2C), with a slightly elevated gephyrin protein level (Supplementary Fig. 2D) likely due to prevention of phosphorylation-dependent degradation by calpain [51]. We recorded mIPSCs from pyramidal neurons in acute slices of the dorsal hippocampal CA1 from WT and *Gphn*^*S268A/S270A*^ mice to evaluate the impact of blocking gephyrin phosphorylation on GABAergic signaling in both sexes. While we did not detect any genotype differences in mIPSC amplitude, we found an increased frequency of mIPSCs in *Gphn*^*S268A/S270A*^ mice, which was significant in female *Gphn*^*S268A/S270A*^ mice compared to WT, indicating potential sex-specific impacts (Fig. 2A). As perisomatic innervation is preferentially detected by whole-cell patch-clamp recordings of the soma [53], we hypothesized that specific enhancement of mIPSC frequency in *Gphn*^*S268A/S270A*^ mice may be explained by altered inputs from the two major inhibitory neuron populations innervating the perisomatic compartment, cholecystokinin (CCK+) and PV+ interneuron subtypes [7]. Using specific markers against CCK+ (cannabinoid receptor 1, CB1 [54]) or PV+ (synaptotagmin 2, Syt2 [10, 55]) axon terminals, we quantified immunoreactive puncta density within the pyramidal cell layer (stratum pyramidale) (Fig. 2B). We found no differences in CB1 puncta density between WT and *Gphn*^*S268A/S270A*^ mice (Supplementary Fig. 2E). However, we detected a sex difference in Syt2+ terminals in WT hippocampi, where female WT mice displayed a lower density of Syt2+ puncta and colocalized Syt2+:GABRG2+ (a post-synaptic marker-GABA_A_ receptor subunit gamma 2) compared to male WT. Intriguingly, *Gphn*^*S268A/S270A*^ mice showed no sex differences in Syt2+ puncta (Fig. 2B). To see if this effect was specific to PV terminals within the pyramidal cell layer, we quantified Syt2+ puncta density in the stratum oriens, finding no differences between the groups (Supplementary Fig. 2G). These data suggest that increased PV neuron inputs onto principal neurons contribute to the increase in functional inhibition in female *Gphn*^*S268A/S270A*^ mice.

The observed sexual dimorphism in Syt2+ terminals could be explained by a reported sex difference in the density of PV interneurons in the dorsal CA1 area of the hippocampus [56, 57]. However, the identification of PV neurons based on immunoreactivity to anti-PV antibodies is confounded by activity-dependent PV expression in the rodent brain [14, 58, 59]. Therefore, to determine whether altered PV neuron density can explain the observed sex and genotype differences in hippocampal Syt2+ terminals, we genetically labeled PV neurons with tdTomato (tdT) (a genetically-encoded fluorophore) by crossing WT or *Gphn*^*S268A/S270A*^ mice to PV-Cre: Ai14-tdTomato mice to achieve Cre-dependent tdT expression. We found that WT females had a lower density of PV neurons compared to WT males (Fig. 2C), mirroring lower WT female Syt2+ terminal density (Fig. 2B). However, *Gphn*^*S268A/S270A*^ mice showed no sex differences in PV neuron density (Fig. 2C). Somatostatin (SST) neurons follow a similar developmental trajectory as PV neurons and are identified by expression of somatostatin protein. Given there were no differences in the density of SST+ somata in the CA1 stratum oriens or stratum pyramidale across all groups (Supplementary Fig. 2F), these data support a specific requirement of gephyrin phosphorylation at S268 and S270 to establish a sex difference in PV-only expressing neuron number and connectivity within the CA1 hippocampal subfield.

### Gephyrin phosphorylation controls parvalbumin protein expression and sex-dependent electrophysiological properties of PV neurons

**Fig. 3:**
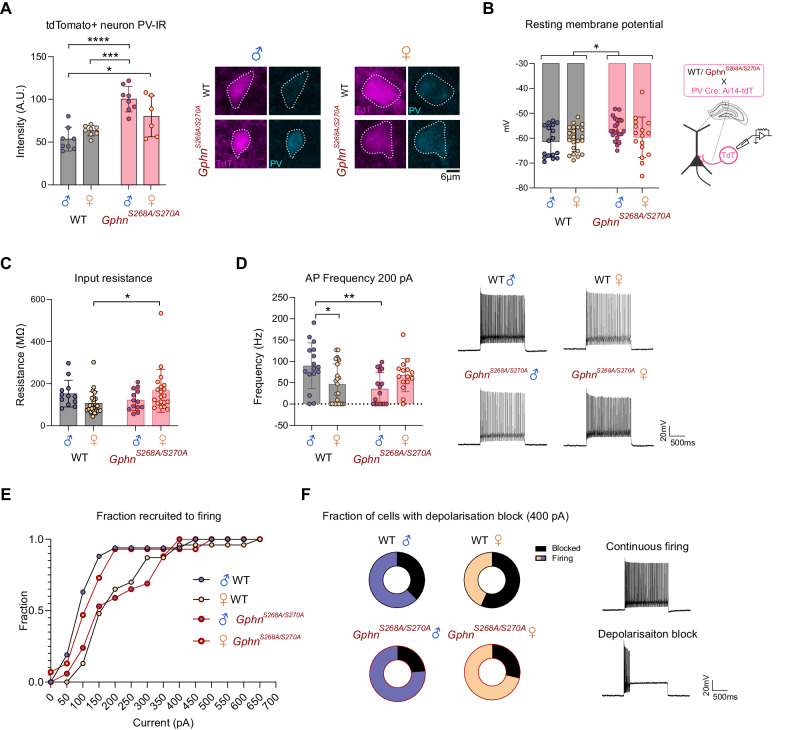
Gephyrin phosphorylation regulates parvalbumin protein expression and sex differences in electrophysiological properties of PV neurons. Male and female WT and *Gphn*^*S268A/S270A*^ mice were crossed to PV-Cre: Ai14-tdT to label PV neurons with tdTomato. **A** tdT+ parvalbumin protein immunoreactivity (PV-IR) staining intensity averages *(Interaction: F(1,26)* = *7.214, p* = *0.0124; genotype: F(1,26)* = *32.14, p* < *0.0001; sex: F(1,26)* = *0.8338, p* = *0.3696)* from 6–10 sections across 6–8 mice per group (left), with representative images (right). **B** The resting membrane potential of tdT+ PV neurons *(Interaction: F(1,77)* = *2.482, p* = *0.1192; sex: F(1,77)* = *0.6202, p* = *0.4334; genotype: F(1,77)* = *4.846, p* = *0.0307)*. **C** Input resistance of tdT+ PV neurons *(Interaction: F(1,65)* = *6.174, p* = *0.0156; sex: F(1,65)* = *0.0007362, p* = *0.9784; genotype: F(1,65)* = *0.6179, p* = *0.4347)*. **D** Action potential (AP) frequency at 200 pA stimulation intensity of tdT+ PV neurons *(Interaction: F(1,68)* = *12.05, p* = *0.0009; sex: F(1,68)* = *0.2681, p* = *0.6063; genotype: F(1,68)* = *2.320, p* = *0.1323)*. **E** Fraction of tdT+ PV neurons spiking at least once during current stimulation at increasing current amplitudes (WT male *n* = 16, female *n* = 23, S268A/S270A male *n* = 17, female *n* = 15). **F** Fraction of tdT+ PV neurons entering depolarization block and stop firing during 400 pA current injections (WT male *n* = 16, female *n* = 23, S268A/S270A male *n* = 17, female *n* = 15). Statistics: Panels **B**–**E** data represent recordings from 17–24 cells/3–4 mice per group. Panels **A**–**D** two-way ANOVA with Sidak post-tests *p < 0.05, **p < 0.01, ***p < 0.001, ****p < 0.0001. Bars, mean ± SD.

Given the altered sex- and phospho-gephyrin-dependent PV neuron density changes observed, we hypothesized that PV neuron function may also be affected. The expression of PV protein can be influenced by neuronal activity/input [60], and may also reflect differences in developmental trajectory [61]. Therefore, we used adult (8–10 wk) WT or *Gphn*^*S268A/S270A*^ mice crossed to PV-Cre: Ai14-tdTomato mice along with PV immunostaining to measure PV immunoreactivity (IR) within tdT+ soma. Strikingly, we found a substantial increase in PV-IR in *Gphn*^*S268A/S270A*^ male PV neurons compared to those of WT males and females (Fig. 3A). This increase relative to WT mice was not significant in female *Gphn*^*S268A/S270A*^ mice, indicating PV protein expression is altered by constitutive blockade of gephyrin phosphorylation in a sex-dependent manner. To directly examine PV neuron function, we used whole-cell patch-clamp electrophysiology to assess intrinsic and firing properties of hippocampal pyramidal cell layer tdT+ neurons in male and female WT and *Gphn*^*S268A/S270A*^ mice. While there was no difference in membrane capacitance across groups (Supplementary Fig. 3A), we detected a slight depolarization of resting membrane potential in *Gphn*^*S268A/S270A*^ PV neurons compared to WT mice (Fig. 3B). This was likely not due to differential health status of *Gphn*^*S268A/S270A*^ PV neurons as input resistance was unchanged in male *Gphn*^*S268A/S270A*^ mice compared to WT (Fig. 3C), and was also significantly increased in female *Gphn*^*S268A/S270A*^ mice compared to WT. There were no differences in action potential (AP) amplitude, half-width, or attenuation across all groups (Supplementary Fig. 3B, C, D). Upon examining PV neuron firing properties in response to increasing current injections (Supplementary Fig. 3E), we found that at low current stimulation amplitudes (e.g., 200 pA), PV neurons of WT females showed a lower average firing frequency compared to those of WT males, while no significant sex differences were observed between male and female *Gphn*^*S268A/S270A*^ mice (*p* = 0.19) (Fig. 3D). This reduced firing frequency in WT female mice is driven in part due to a smaller fraction of PV neurons being recruited to firing at lower stimulation intensities, requiring larger current amplitudes to elicit firing (Fig. 3E). By contrast, female *Gphn*^*S268A/S270A*^ PV neurons were recruited to firing akin to WT males, while male *Gphn*^*S268A/S270A*^ PV neurons more closely resembled WT females. When examining higher stimulation current amplitudes (e.g., 400 pA), we observed that WT female PV neurons were more likely to enter depolarization block compared to WT male neurons. This sex difference was not observed in *Gphn*^*S268A/S270A*^ PV neurons (Fig. 3F). Taken together, these electrophysiological data suggest that gephyrin phosphorylation differentially affects PV neuron activity and intrinsic properties in WT males and females, with likely consequences for circuit function.

### Gephyrin phosphorylation is required for the convergent performance of hippocampal-dependent cognition in males and females

**Fig. 4:**
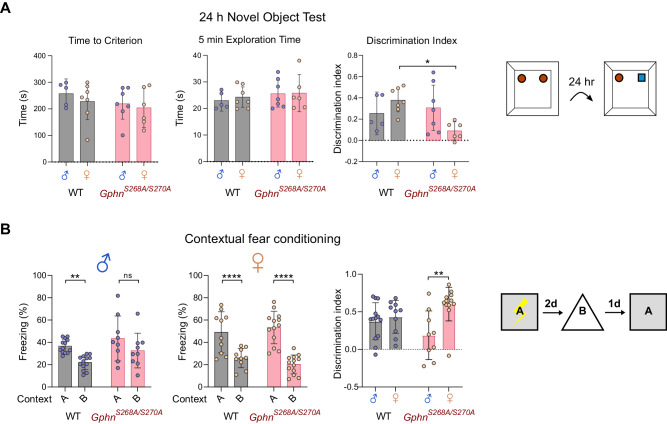
Constitutive blockade of gephyrin S268/S270 phosphorylation results in sex-specific hippocampal-dependent memory deficits. **A** In the novel object test, mice were allowed to explore two objects and exposed 24 h later to one identical object and one novel object. Left panel: time to reach 20 s of exploration time (time to criterion) *(Interaction: F(1,21)* = *0.07123, p* = *0.7922; sex: F(1,21)* = *0.7263, p* = *0.4037; genotype: F(1,21)* = *1.383, p* = *0.2527)*.; middle panel: the total exploration time *(Interaction: F(1,21)* = *0.07502, p* = *0.7868; sex: F(1,21)* = *0.1348, p* = *0.7172; genotype: F(1,21)* = *1.005, p* = *0.3275)*; right panel: discrimination index (positive index indicating novel object preference) *(Interaction: F(1,21)* = *6.929, p* = *0.0156; sex: F(1,21)* = *0.5055, p* = *0.4849; genotype: F(1,21)* = *3.314, p* = *0.0830)*. **B** In contextual fear conditioning, mice were shocked in context A, and compared for percent time spent freezing in context A or context B (left panels) for males (Context*genotype: F(1,20) = *0.3274, p* = *0.5736; context: F(1,20)* = *14.23, p* = *0.0012; genotype: F(1,20)* = *4.364, p* = *0.0497; subject: F(1,21)* = *1.526, p* = *0.1763)*, female (Context*genotype: F(1,20) = *0.2471, p* = *0.1127; context: F(1,21)* = *88.29, P* < *0.0001; genotype: F(1,21)* = *0.02413, p* = *0.8780; subject: F(1,21)* = *2.371, p* = *0.0270)* differential freezing was compared using a discrimination index (right panel) *(Interaction: F(1,41)* = *5.532, p* = *0.0236; sex: F(1,41)* = *9.620, p* = *0.0035; genotype: F(1,41)* = *0.01023, p* = *0.9199)*. Data represent individual mice, *n* = 5–13 per group. Statistics: Panel **A** – Two-way ANOVA with Sidak post-tests. Panel **B** – Two-way repeated measures ANOVA. *p < 0.05, **p < 0.01, ****p < 0.0001. Bars, mean ± SD.

Based on the sex-dependent disruption of PV neuron density and functional changes in the *Gphn*^*S268A/S270A*^ phospho-null mice, we hypothesized that these mice may show alterations in hippocampal-dependent cognitive processes. Therefore, we assessed both male and female WT and *Gphn*^*S268A/S270A*^ adult (10–12-week-old) mice for hippocampal-dependent long-term memory function using novel object recognition, object location, and contextual fear memory tests. The novel object recognition (NOR) and object location tests assess long-term memory by taking advantage of a mouse’s preference for exploring novel objects or object locations 24 h after prior exposure to two identical objects. WT mice male and female mice showed the same (convergent) performance in either memory task. However, *Gphn*^*S268A/S270A*^ showed divergent performance that was task-specific. Female *Gphn*^*S268A/S270A*^ mice showed a deficit in object recognition memory compared to female WT mice (Fig. 4A). No sex or genotype differences were observed in 24 h object location memory (Supplementary Fig. 4A). In a contextual memory test assessing hippocampal-dependent fear memory, mice learn freezing behavior to a shock in context A, and must discriminate this from context B (Supplementary Fig. 4D). While WT male and female mice can discriminate between these contexts, male *Gphn*^*S268A/S270A*^ mice did not, and additionally showed reduce discrimination abilities compared to *Gphn*^*S268A/S270A*^ females (Fig. 4B), suggesting impaired contextual memory in *Gphn*^*S268A/S270A*^ males. These sex and *Gphn*^*S268A/S270A*^-dependent memory changes are likely not due to differences in anxiety or locomotor deficits as there were no genotype differences in an elevated plus maze (Supplementary Fig. 4B) or open field test (Supplementary Fig. 4C). It should be noted that for open field center time, a significant sex effect was detected (without interaction effect) in WT mice, with females showing an increase in center time. Taken together, gephyrin phosphorylation is required for convergent hippocampal cognition in adult male and female mice, with task-specific impacts on hippocampal memory in male or female mice when phosphorylation is constitutively blocked.

### Gephyrin phosphorylation generates postnatal sexual dimorphism in hippocampal interneuron development

**Fig. 5:**
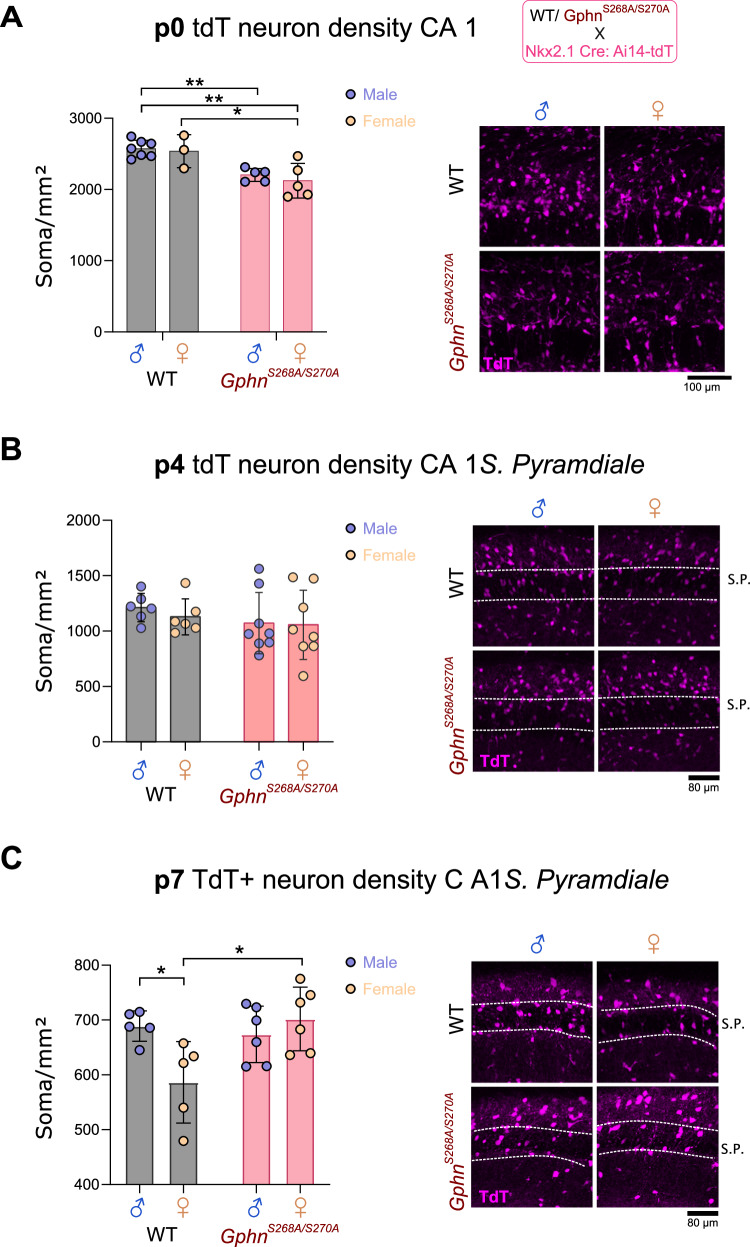
Sex differences in putative PV neurons emerge during early postnatal development and are dependent on gephyrin phosphorylation. Male and female WT and *Gphn*^*S268A/S270A*^ mice were crossed to Nkx2.1-Cre: Ai14-tdT to label putative hippocampal PV neurons with tdTomato at p0, p4, and p7. **A** tdT+ soma density within CA1 at p0 *(Interaction: F(1,16)* = *0.08385, p* = *0.7759; genotype: F(1,16)* = *24.30, p* = *0.0002; sex: F(1,16)* = *0.5384, p* = *0.4737)*. **B** tdT+ soma density within CA1 stratum pyramidale at p4 *(Interaction: F(1,24)* = *0.1332, p* = *0.7183; genotype: F(1,24)* = *0.2843, p* = *0.5988; sex: F(1,24)* = *1.292, p* = *0.2669)*. **C** tdT+ soma density within CA1 stratum pyramidale at p7 *(Interaction: F(1,18)* = *7.785, p* = *0.0121; sex: F(1,18)* = *2.054, p* = *0.1690; genotype: F(1,18)* = *4.528, p* = *0.0474)*. Data represent individual averages across 6–12 sections. Statistics: All panels – two-way ANOVA with Sidak post-tests *p < 0.05. *p < 0.01. Bars, mean ± SD.

Electrophysiological alteration of PV neuron function in male and female adult *Gphn*^*S268A/S270A*^ mice could derive from blocked phosphorylation in adulthood, however, changes in PV neuron density likely arise earlier during development. There is currently no available tool to specifically label PV neurons before the second postnatal week, due to the late onset of PV protein expression. Therefore, to examine whether gephyrin phosphorylation at S268/S270 results in sexually dimorphic PV density changes during early development, we used WT or *Gphn*^*S268A/S270A*^ mice crossed to Nkx2.1-Cre: Ai14-tdTomato mice to label medial ganglionic eminence (MGE)-derived interneurons with tdTomato, which include both PV-type and SST-type interneurons [43]. We quantified tdT+ somata in the CA1 area of the hippocampus at p0 (when developing MGE-derived neurons have mostly migrated to the hippocampal formation), at p4, and at p7 (close to the peak of postnatal interneuron apoptosis). Our analysis at p4 and p7 focused on the pyramidal cell layer where a strong enrichment of putative PV neurons is expected. At p0, no sex differences were evident in tdT+ interneuron number in CA1, although a genotype effect was observed, where *Gphn*^*S268A/S270A*^ male and female mice displayed fewer tdT+ somata compared to WT (Fig. 5A.). At P4, there was no genotype difference in tdT+ neuron density (Fig. 5B). As SST protein expression was readily detectable after p4, we were able to quantify tdT+/SST+ soma density at p7, discriminating effects on SST versus putative PV neurons. By P7, adult-like sex differences in tdT+ neuron density were detected between WT males and females, which was not present between *Gphn*^*S268A/S270A*^ male and female mice (Fig. 5C). There were no differences in SST neuron density (tdT+/SST+) in the stratum radiatum or pyramidale (Supplementary Fig. 5A), suggesting that sex differences in MGE-derived interneuron number in the stratum pyramidale may be specific to PV neurons, and emerge early during postnatal development.

### Gephyrin phosphorylation establishes sexual dimorphism in developing putative PV neuron electrophysiological properties and transcriptomic profiles

**Fig. 6:**
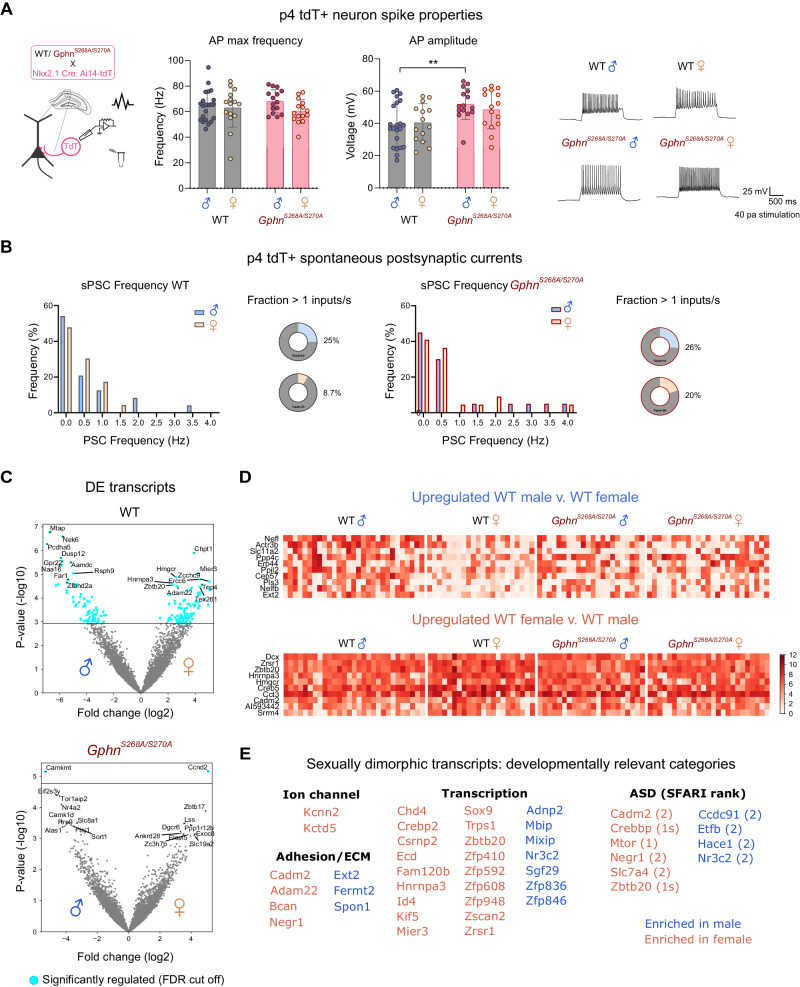
Patch-sequencing reveals that gephyrin phosphorylation establishes electrophysiological properties and sexual dimorphism of transcriptomic state in developing putative PV CA1 neurons at p4. Male and female WT and *Gphn*^*S268A/S270A*^ mice were crossed to Nkx2.1-Cre: Ai14-tdT mouse lines to label putative hippocampal PV neurons with tdTomato at p4 for patch-clamp electrophysiology and single neuron sequencing. **A** Action potential frequency (left) *(Interaction: F(1,63)* = *1.398, p* = *0.2415; sex: F(1,63)* = *2.442, p* = *0.1232; genotype: F(1,63)* = *0.02019, p* = *0.8875)*, action potential amplitude (right) *(Interaction: F(1,62)* = *0.6012, p* = *0.4411; sex: F(1,62)* = *0.1302, p* = *0.7195; genotype: F(1,62)* = *12.20, p* = *0.0009)* and representative traces of tdT+ putative PV neurons from dorsal hippocampal slices in the stratum pyramidale at p4. **B** Spontaneous post-synaptic current (PSC) input frequency histogram and the fraction of neurons receiving inputs at >1 Hz (WT male *n* = 24, female *n* = 23, S268A/S270A male *n* = 19, female *n* = 20). **C** Differentially expressed (DE) transcripts between WT and *Gphn*^*S268A/S270A*^ male and female mice, transcripts in blue represent significant DE transcripts. **D** Expression heat map of the top 10 most DE sexually dimorphic transcripts up or downregulated in WT male v. WT female mice. **E** Developmentally relevant categorization of WT sexually dimorphic transcripts. Blue: transcripts elevated in males; orange: transcripts elevated in females. Statistics: Panels A + B: data represent individual cells - WT male *n* = 22 cells from 8 pups, WT female *n* = 15 cells from 7 pups, *Gphn*^*S268A/S270A*^ male *n* = 5 cells from 9 pups, *Gphn*^*S268A/S270A*^ female *n* = 15 cells from 10 pups. Panel **C**–**E** – data represent individual cells: WT male *n* = 29 cells from 8 pups, WT female *n* = 22 cells from 7 pups, *Gphn*^*S268A/S270A*^ male *n* = 22 cells from 9 pups, *Gphn*^*S268A/S270A*^ female *n* = 25 cells from 10 pups. Two-way ANOVA with Sidak post-tests **p < 0.01. Panel **C** see “Methods” section for detailed statistical information. Bars, mean ± SD.

Both activity-dependent and transcriptional programs influence interneuron maturation during postnatal development. Hence, to understand whether gephyrin phosphorylation impacts PV neurons during a critical developmental time window, we used patch-sequencing [62–64] to define the electrophysiological properties and transcriptional profile of putative PV neurons at p4. This serves as an ideal time point to identify putative PV neurons, because MGE-derived neurons have already migrated to their correct layer and start to form connections with surrounding neurons [7], but have not yet developed sex differences in PV neuron density (see Fig. 5C). Using Nkx2.1-Cre: Ai14-tdTomato mice crossed to WT or *Gphn*^*S268A/S270A*^ mice, we recorded electrophysiological properties and single-cell transcriptional profiles of MGE-derived tdT+ cells in the CA1 pyramidal cell layer, where developing PV neurons are enriched. Whole-cell recordings of p4 tdT+ neurons revealed no difference in the resting membrane potential (Supplementary Fig. 6A), input resistance (Supplementary Fig. 6B) or capacitance (Supplementary Fig. 6C) across all groups. When examining spiking properties of putative PV neurons, we found no difference in maximum firing frequency (Fig. 6A). However, male but not female *Gphn*^*S268A/S270A*^ neurons had a significantly higher spike amplitude (Fig. 6A), with no difference in action potential half-width (Supplementary Fig. 6D) or attenuation (Supplementary Fig. 6E). We additionally recorded spontaneous post-synaptic currents (sPSCs) to assess whether these neurons are differentially targeted by developing inputs. There were few sPSCs detected in general at this age, however, qualitatively we found a smaller fraction of WT female tdT+ neurons received inputs at >1 Hz compared to those of WT males, while there was no qualitative difference between male and female *Gphn*^*S268A/S270A*^ mice (Fig. 6B). To check whether developing hippocampal networks show sex differences in neuron activation at p4, we quantified cFOS+ cell density in the hippocampal CA1. Remarkably, WT females showed a significantly lower cFOS+ cell density at p4 compared to WT males (Supplementary Fig. 6F). However, female *Gphn*^*S268A/S270A*^ cFOS+ cell density was higher than WT, without differences compared to WT female or *Gphn*^*S268A/S270A*^ male mice, suggesting that network activation may be equalized by blocking gephyrin phosphorylation. Taken together, these data indicate that WT female PV neurons may have altered network input at p4 compared to WT male PV neurons. Blocking gephyrin phosphorylation normalizes these sex differences, and enhances spike amplitude in males, potentially impacting sex-dependent circuit development into adulthood.

**Fig. 7:**
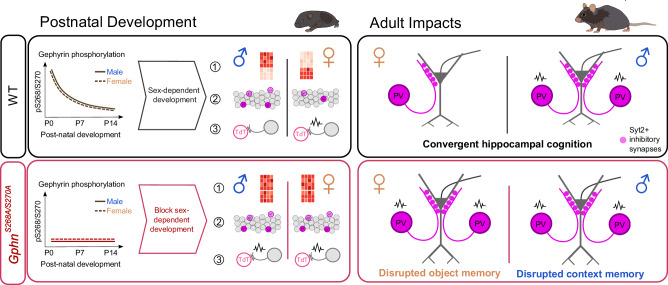
Summary of sex-specific impacts of gephyrin phosphorylation: postnatal development and adult impact on PV neurons and hippocampal function. Postnatal development: In WT mice, gephyrin phosphorylation levels decline during the first two postnatal weeks in males and females. This phosphorylation impacts gephyrin’s influence on normal sexually dimorphic development (e.g., via post-synaptic regulation of GABAergic signaling, or regulation of gephyrin’s non-synaptic functions) to establish sexual dimorphism in putative PV neuron (1) transcriptional state, (2) density within the stratum pyramidale, and (3) electrophysiological properties and input. However, blocking this developmental phosphorylation in *Gphn*^*S268A/S270A*^ mice prevents the development of these sex differences. Adult impacts: Sex differences in PV neuron density that emerge postnatally are preserved in adult WT mice, where PV neuron electrophysiological function and inputs are different between males and females. Despite these PV neuron sex differences, hippocampal function is convergent. In *Gphn*^*S268A/S270A*^ mice, PV neuron sex differences are blocked and associated with disrupted hippocampal cognition in males (contextual memory) and females (object memory). Taken together, gephyrin phosphorylation establishes sex differences in PV neuron development and adult function, disruption of which leads to sex-specific deficits in cognition.

We subsequently analyzed transcriptomic differences from the aspirated cytosol of tdT+ neurons and used next-generation RNA sequencing to determine sex and genotype differences in transcriptional profile. Only the cells that passed QC analysis were included (see “Methods” section *WT male n* = *29 cells, WT female n* = *22 cells, Gphn*^*S268A/S270A*^ *male n* = *22 cells, Gphn*^*S268A/S270A*^ *female n* = *25 cells*). Considering transcripts detected in all groups, WT males and females showed 140 significantly differentially expressed (DE) transcripts (*Log*_*2*_*(FC)* > *1, FDR* < *0.05*), while these sex differences were essentially abolished in *Gphn*^*S268A/S270A*^ mice (*only two DE transcripts*) (Fig. 6C). Strikingly, amongst the top 10 most DE transcripts between male and female WT mice, no differences were observed between male and female *Gphn*^*S268A/S270A*^ mice (Fig. 6C, D). Amongst the differentially expressed transcripts between male and female WT mice, two transcripts were linked to ion channel function, with many transcripts coding for transcription factors, adhesion, and extracellular matrix (ECM) proteins. Several of these sex-regulated genes are also associated with autism spectrum disorders according to SFARI gene classification (Fig. 6E). Together, these data suggest that gephyrin phosphorylation regulates putative PV neuron signaling and physiological properties prior to major synaptogenesis, indicating disruption of gephyrin phosphorylation during early postnatal development may profoundly affect the sexually dimorphic development of PV neuron density, connectivity, and function into adulthood (Fig. 7).

## Discussion

### Developmental relevance of gephyrin phosphorylation at S268 and S270

In this study, we discovered a dramatic developmental decline of gephyrin phosphorylation at S268 and S270 in WT mice occurring prior to network maturation. Constitutive blockade of gephyrin phosphorylation in *Gphn*^*S268A/270A*^ mice abolished sex differences in parvalbumin neuron density and function starting from early postnatal development. This suggests an unappreciated role of gephyrin phosphorylation in establishing inhibitory connectivity by controlling the number/function of PV neurons in the hippocampus. Interneurons form functional synapses from p0 onwards that mature and stabilize over time [7]. ERK1/2 phosphorylation of gephyrin at S268 triggers calpain-dependent degradation and reduction in gephyrin clustering [38] and GSK3β phosphorylation of S270 reduces gephyrin cluster size [51]. Therefore, elevated postnatal gephyrin S268/S270 phosphorylation could promote destabilizing conditions during synapse pruning, while reduced phosphorylation later in development facilitates synapse stabilization and maturation. Gephyrin is expressed in neurons and glia serving both synaptic and non-synaptic functions [65]. While we used immunoblotting to assess gephyrin phosphorylation at the tissue level, we currently lack the tools to define phosphorylation at the neuron-specific and synapse-specific levels due to technical limitations of phospho-antibodies for immunofluorescence. Development of new tools is therefore required to determine if gephyrin phosphorylation is differentially regulated in different cell types during development (e.g., PV neuron versus SST type) to achieve PV neuron-specific effects.

### Preventing sex differences in PV neurons is associated with disrupted hippocampal function

Defining the sex-specific regulation and function of PV neurons is integral to understanding their dysfunction in sex-biased brain disorders. In C57B6/J mice, male-female differences in hippocampal neuron density have been documented, but the underlying signaling has not been studied [56, 57, 66]. PV neurons display sex-specific connectivity in the ventral subiculum [67], and sex differences in PV neuron density have been noted in the context of resilience to stressors [14, 59], oxidative stress [68], and monogenic disease-related gene knockout mice [2, 9, 16, 68]. Indeed, early life stress specifically affects female PV neuron density in the orbitofrontal cortex, comorbid with deficits in reversal learning [69]. Our data detected sex differences in the density of PV neurons within the stratum pyramidale, representing several PV neuron subtypes: basket cell (BC), bistratified cell (BS), and axo-axonic cell (AAC). PV-BS cells co-express somatostatin, and we did not see differences in somatostatin+ neurons or Syt2+ terminals in the stratum oriens. Given the relatively low percentage of PV-AAC compared to BC and the lack of effect on putative PV-BS neurons, it is likely that PV-BCs are particularly affected by gephyrin phosphorylation. Patch-sequencing and morphological reconstruction of PV neurons could confirm the molecular and anatomical subtypes of PV neurons most affected. Multiple functional populations of adult PV neurons have been identified, including a subset that enters premature depolarization block [70], although the sex-dependency of this is unclear. We found that in WT mice, intrinsic membrane properties and action potential amplitude do not differ between sexes. However, at low stimulation intensities, fewer WT female PV neurons are recruited to firing and additionally are more likely to enter depolarization block. Similar sex differences were not observed in *Gphn*^*S268A/S270A*^ mice. PV neuron temporal precision is key for controlling hippocampal oscillations thought to be important for memory [71]. Males and females are known to use distinct strategies for hippocampal memory encoding [72]. Therefore, the similar performance of male and female WT mice in three hippocampal-dependent memory tests indicates that males and females under typical developmental conditions may employ circuits with differential involvement of PV neuron function. A differential reliance on PV neurons could help explain the dysfunction observed for *Gphn*^*S268A/S270A*^ females in the novel object test and *Gphn*^*S268A/S270A*^ males in the contextual fear memory test. Specifically examining the role of gephyrin phosphorylation for PV neuron-intrinsic function in hippocampal memory will require new models to control gephyrin phosphorylation in a cell-type-specific manner.

### The link between gephyrin phosphorylation and interneuron development

We show that gephyrin phosphorylation influences PV neurons’ sexually dimorphic features including cell density, electrophysiological function, and transcriptional profile by p7. Mechanistically, this could occur through gephyrin’s synaptic or non-synaptic roles impacting PV neurons before or during early postnatal development. During development, synaptic connectivity and activity impact immature PV neurons, where increased connectivity promotes neuron integration and survival [29]. GABAergic input during early postnatal development is largely excitatory, driving calcium entry required for cell survival and maturation [30, 31, 33, 34, 73]. We found that constitutively preventing gephyrin phosphorylation blocks sexual dimorphism of putative PV neuron density in WT mice before p7, and blocks sex differences in transcriptomic profile by p4. The developmental decline of excitatory GABA signaling is region-dependent [74], occurring earlier in female hippocampi [19]. Therefore, blocking gephyrin phosphorylation in females may raise GABAergic excitatory drive to a similar level as in males for precocious neuron and circuit maturation. Indeed, we found that WT female hippocampi showed fewer cFOS+ cells compared to WT males, while blocking gephyrin phosphorylation prevented these sex differences and increased hippocampal activation in *Gphn*^*S268A/S270A*^ females. Additionally, a higher fraction of female *Gphn*^*S268A/S270A*^ neurons received spontaneous PSCs over 1 Hz compared to female WT, suggesting more functional input, though the current sample size precludes quantitative analysis. At the transcriptional level, p4 levels of doublecortin (*Dcx*, an immature neuron marker) were elevated in WT female interneurons compared to males, and expression of neurofilament light chain (*Nefl*, a mature neuron marker), was higher in WT males compared to females. However, these sex differences were not observed in *Gphn*^*S268A/S270A*^ mice, suggesting that gephyrin phosphorylation could tune developing hippocampal inhibition to establish sexually dimorphic PV neuron developmental trajectories.

In addition to the above synaptic mechanisms, gephyrin phosphorylation’s effect on its non-synaptic functions could additionally impact PV neuron development. Gephyrin is an enzyme in the molybdenum cofactor (MOCO) biosynthesis pathway, important for sulfite oxidase function [65, 75, 76]. In the nervous system this role is accomplished by a specific non-synaptic gephyrin splice isoform expressed in glia [65] without a clear link to PV- or sex-specific functionality. Moreover, our mice did not display the severe symptoms of MOCO deficiency. Gephyrin also scaffolds mTOR signaling complexes in neurons [77, 78], however, the involvement of phosphorylation in this process is not known [79, 80]. A comprehensive analysis of gephyrin’s protein-protein interactions in the brain revealed transcription factors and splicing complex components as potential “moonlighting” regulatory functions of gephyrin [81] that could regulate sex-dependent transcriptional profiles. However, the precise link between gephyrin phosphorylation at S268 and S270 to these non-synaptic functions requires specific examination to understand any contribution to interneuron development. Regardless of the proximal cause, the impact of gephyrin phosphorylation on sex differences in developing interneuron transcriptomes likely has a bearing on adult functionality, considering the large number of transcription factors, ECM, and adhesion proteins showing sex differences in WT mice (e.g. brevican (*Bcan*), a core component of PV neuron perineuronal nets [82]). Moreover, the identification of multiple SFARI-ranked genes in this dataset warrants future research into how the sex-specific expression of these transcripts influences interneuron dysfunction in ASDs.

### Relevance of gephyrin phosphorylation in disease

To our knowledge, no one has examined how developmental synaptic protein phosphorylation controls neuron or circuit maturation. This may be due to a historical study of synaptic protein posttranslational modifications in the context of synaptic plasticity. Large-scale sequencing efforts have matched human genomic variants to mRNA expression levels and associated phenotypes. However, linking phosphorylation patterns to phenotypes in humans has not been performed, as this would require tissue-specific phospho-proteomic profiling. Partial genomic gephyrin deletions are associated with ASDs and schizophrenia [40], indicating pathological links to gephyrin disruption. As of 2024, missense variants of human gephyrin serine 270 (ClinVar VCV000423239.4) have been identified but not functionally annotated. Pathogenic differences in phosphorylation of S268 and S270 would be expected to result in similar phenotypes to a genetic phospho-null model, and indeed the kinases that target these sites (ERK1/2 and GSK3β) show altered signaling in models of psychiatric disorders [24, 28, 83, 84].

In conclusion, phosphorylation of gephyrin at S268 and S270 is associated with directing sex differences in hippocampal PV neuron development and function, and disrupting this phosphorylation causes sex-specific impacts on hippocampal cognition (Fig. 7). Thus, gephyrin phosphorylation acts at two levels to control inhibitory function: 1) by regulating GABAergic inhibitory post-synaptic function (as established by previous studies [38, 39]), and 2) by tuning the number and function of presynaptic PV neurons (as explored in this study). Moreover, this description of sexually dimorphic PV neuron development and function despite convergent cognition highlights that males and females may use distinct circuit/signaling strategies to achieve equal function, and may help to explain differential susceptibility to diseases that present with inhibitory hypofunction. The sex-specific impacts of disrupting this phosphorylation highlight the need to characterize kinase activity and substrate modification in males and females during brain development, bridging the current gap in our knowledge of what contributes to sexual dimorphism in brain function and dysfunction. Understanding the contributions of phosphorylation to brain development may additionally explain the “environment factor” on the frequency and phenotypic penetrance of sex-biased psychiatric and developmental brain disorders.

## Supplementary information

Supplementary Figures

Supplementary Data file

## Data Availability

The authors declare no restrictions on data availability.
