# Supplementary material for: Gephyrin phosphorylation facilitates sexually dimorphic development and function of parvalbumin interneurons in the mouse hippocampus
Source: Mol Psychiatry. 2024 Mar 19;29(8):2510–26. doi: 10.1038/s41380-024-02517-5 (PMC11412903; doi:10.1038/s41380-024-02517-5)
Supplement: Supplementary file 1 — Supplementary Figures [file 41380_2024_2517_MOESM1_ESM.pdf]

Supplementary figure legends

Supplemental Figure 1. **Gephyrin phosphorylation is regulated between adult male and female mice but not during the estrus cycle. A)** Immunoblot of total gephyrin and phosphorylated gephyrin at S268 and S270 from hippocampal lysates of adult male and female WT mice. **B)** Immunoblotting of total (*F(3, 20)=0.7907, p=0.5133*) or phospho-S268 (*F(3, 20)=0.7351, p=0.5433*) and phospho-S270 (*F(3, 20)=0.4006, p=0.7541*) at different estrous stages. **Statistics**: each data represents lysates from individual mice, n=4-6 per group. Phospho-gephyrin levels are represented relative to total gephyrin. Panel A: two-way t-test; panel B: – One-way ANOVA with post-test comparing all groups. ** p<0.01,. Bars, mean ± SD.

Figure 2 Supplement. Characterization of ***Gphn***$^{S268A/S270A}$ **mice**. **A)** Schematic of *Gphn*$^{S268A/S270A}$ constitutive phospho-null mice. **B)** No difference in the genotype ratio of mice born to heterozygous (het) parents in both males (*Chi*^{2}*=0.200, p=0.9048*), females (*Chi*^{2}*=0.000, p=1.000*), or pooled across sex (*Chi*^{2}*=0.125, p=0.9394*) from 11 litters, 6-10 pups/litter. **C)** No genotype difference in body weight in males (*F=0.8646, p=0.4271*) or females (*F=2.147, p=0.1387*), or brain weight in males (*F=1.067, p=0.4015*) or females (*F=2.473, p=0.1141*) from 3-24 mice per group. **D)** Immunoblot of total gephyrin protein in *Gphn*$^{S268A/S270A}$ male (*F(2, 18)=4.421, p=0.0274*) and female (*F(2, 6)=6.393, p=0.0326*) hippocampi compared to WT (2-7 mice/group). **E)** Density of CB1+ basket cell terminals in the stratum pyramidale (*Interaction: F(1,24)=2.265, p=0.1454; genotype: F(1,24)=0.8427, p=0.3678; sex: F(1,24)=0.5453, p=0.4674*) from 5-8 mice/group. **F)** Somatostatin (SST+) soma density in the CA1 hippocampal stratum oriens (S.O.) (*Interaction: F(1,23)=0.5027, p=0.4854; genotype: F(1,23)=0.6938, p=0.4134; sex: F(1,23)=2.906, p=0.1017*) and stratum pyramidale (S.P.) (*Interaction: F(1,23)=0.08147, p=0.7779; genotype: F(1,23)=0.3323, p=0.5699; sex: F(1,23)=2.813, p=0.1071*). **G)** Syt2+ puncta density in the stratum oriens (*Interaction: F(1,19)=2.069, p=0.1666; genotype: F(1,19)=0.5577, p=0.4643; sex: F(1,19)=0.9459, p=0.3430*). Data represent averages across 6-10 sections per individual, 5-8 mice per group. **Statistics**: Panel B – Chi-squared test; Panels C+D: One-way ANOVA with post-test comparing all groups; Panels E, F: Two-way ANOVA with Sidak post-tests. * p<0.05. Bars, mean ± SD.

Figure 3 Supplement. Gephyrin phosphorylation regulation of PV neuron electrophysiological properties. Male and female WT and *Gphn*$^{S268A/S270A}$ mice were crossed to PV-Cre: Ai14-tdT to label PV neurons with tdTomato to identify PV neurons for patch clamp electrophysiology. **A)** No differences in PV neuron capacitance (*Interaction: F(1,64)=2.251, p=0.1385; genotype: F(1,64)=0.09933, p=0.7537; sex:*

F(1,64)=0.2014, p=0.6551), **B)** action potential amplitude (*Interaction: F(1,77)=0.4608, p=0.4993; genotype: F(1,77)=0.6135, p=0.4359; sex: F(1,77)=0.1195, p=0.7305*), **C)** action potential half width (*Interaction: F(1,77)=0.09472, p=0.7591; genotype: F(1,77)=0.02846, p=0.8665; sex: F(1,77)=0.8681, p=0.3546*), and **D)** action potential attenuation (*Interaction: F(1,75)=0.5657, p=0.4543; genotype: F(1,75)=2.013, p=0.1601; sex: F(1,75)=0.06807, p=0.7949*). **E)** Maximum PV firing frequency by stimulation intensity (pA). **Statistics**: All panels: data represent recordings from 17-24 cells/3-4 mice per group; Panels A-D: two-way ANOVA with Sidak post-tests. Bars, mean ± SD.

Figure 4 Supplement. Extended behavioral analysis of WT and Gphn$^{S268A/S270A}$ male and female mice. **A)** Object location test: mice were given spatial cues in the arena while exploring two objects, one of which was moved to the opposite corner 24 hours later. Time to criterion (*Interaction: F(1,36)=0.5267, p=0.4727; sex: F(1,36)=1.319, p=0.2584; genotype F(1,36)=0.1900, p=0.6655*); 5 minute exploration time (*Interaction: F(1,41)=0.01305, p=0.9096; sex: F(1,41)=0.05383, p=0.8177; genotype F(1,41)=0.9184, p=0.3435*); discrimination index (*Interaction: F(1,37)=0.6305, p=0.4322; sex: F(1,37)=0.2441, p=0.6242; genotype F(1,37)=1.516, p=0.2259*). **B)** Elevated plus maze: time in open arms (*Interaction: F(1,25)=0.001773, p=0.9668; sex: F(1,25)=0.8125, p=0.3760; genotype F(1,25)=0.1589, p=0.6935*); time in closed arms (*Interaction: F(1,25)=0.004003, p=0.9501; sex: F(1,25)=0.5176, p=0.4785; genotype F(1,25)=0.3010, p=0.5882*), and time spent in the center (*Interaction: F(1,25)=0.03282, p=0.8577; sex: F(1,25)=0.1115, p=0.7413; genotype F(1,25)=0.3698, p=0.5486*) from n=5-8 mice/group. **C)** Mice were monitored in a 15-minute open field test to measure total distance moved (*Interaction: F(1,34)=0.4222, p=0.5202; sex: F(1,34)=3.920, p=0.0558; genotype F(1,34)=0.08455, p=0.7730*), center time (*Interaction: F(1,36)=0.4489, p=0.5072; sex: F(1,36)=11.69, p=0.0016; genotype F(1,36)=0.8482, p=0.3634*), and average speed (*Interaction: F(1,34)=0.3784, p=0.5426; sex: F(1,34)=0.1529, p=0.6982; genotype F(1,34)=3.554, p=0.0680*) from n=7-11 mice/group. **D)** Male and female mouse freezing during learning trials (T) 1-5 of the contextual fear memory test. Data represent individual mice, n=5-13 per group. **Statistics**: Panels A - D – two-way ANOVA with Sidak post-tests * p<0.05. Bars, mean ± SD.

Figure 5 Supplement. **No sex differences in SST hippocampal neuron density.** Male and female WT and *Gphn$^{S268A/S270A}$* mice were crossed to Nkx2.1-Cre: Ai14-tdT to label putative hippocampal PV neurons with tdTomato at p7. **A**) tdT+/SST+ cell density within CA1 stratum oriens (*Interaction: F(1,18)=0.1939, p=0.6650; genotype: F(1,18)=0.2036, p=0.6572; sex: F(1,18)=0.6427, p=0.4332*) and stratum pyramidale (*Interaction: F(1,19)=0.7321, p=0.4029; genotype: F(1,19)=0.6181, p=0.4414; sex: F(1,19)=0.2385,*

p=0.6309). Data represent individual averages across 6-12 sections. **Statistics**: Two-way ANOVA with Sidak post-tests. Bars, mean ± SD.

Figure 6 Supplement. Extended electrophysiological analysis of putative PV interneurons at p4 and hippocampal cFOS expression. Male and female WT and *Gphn*$^{S268A/S270A}$ mice were crossed to Nkx2.1-Cre: Ai14-tdT to label putative hippocampal PV neurons with tdTomato at p4 for patch-sequencing of dorsal CA1 pyramidal layer interneurons. **A)** tdt+ neuron resting membrane potential (*Interaction: F(1,81)=0.2004, p=0.6556; sex: F(1,81)=0.06568, p=0.7984; genotype: F(1,81)=0.5556, p=0.4582)*, **B)** Input resistance (*Interaction: F(1,66)=0.4758, p=0.4928; sex: F(1,66)=0.9609, p=0.3305; genotype: F(1,66)=0.7460, p=0.3903)*, **C)** Capacitance (*Interaction: F(1,71)=0.7696, p=0.3833; sex: F(1,71)=0.004031, p=0.9496; genotype: F(1,71)=0.05872, p=0.8092*), **D)** Action potential half width (*Interaction: F(1,61)=2.967, p=0.0900; sex: F(1,61)=0.1275, p=0.7223; genotype: F(1,61)=0.1907, p=0.6639*) and **E)** attenuation (*Interaction: F(1,59)=0.0230, p=0.8872; sex: F(1,59)=1.621, p=0.208; genotype: F(1,59)=4.464, p=0.0389)*. **F)** cFOS+ cell density in the hippocampal CA1 at p4 (*Interaction: F(1,12)=8.525, p=0.0128; sex: F(1,12)=6.256, p=0.0279; genotype: F(1,12)=6.064, p=0.0299).* **Statistics**: Panels A-E: data represent individual cells - WT male n=22 cells from 8 pups, WT female n=15 cells from 7 pups, *Gphn*$^{S268A/S270A}$ male n=5 cells from 9 pups, *Gphn*$^{S268A/S270A}$ female n=15 cells from 10 pups. Panel F: Data represent individual averages (6-8 sections/mouse, n=4 mice per group). All panels: Two-way ANOVA with Sidak post-tests. Bars, mean ± SD.

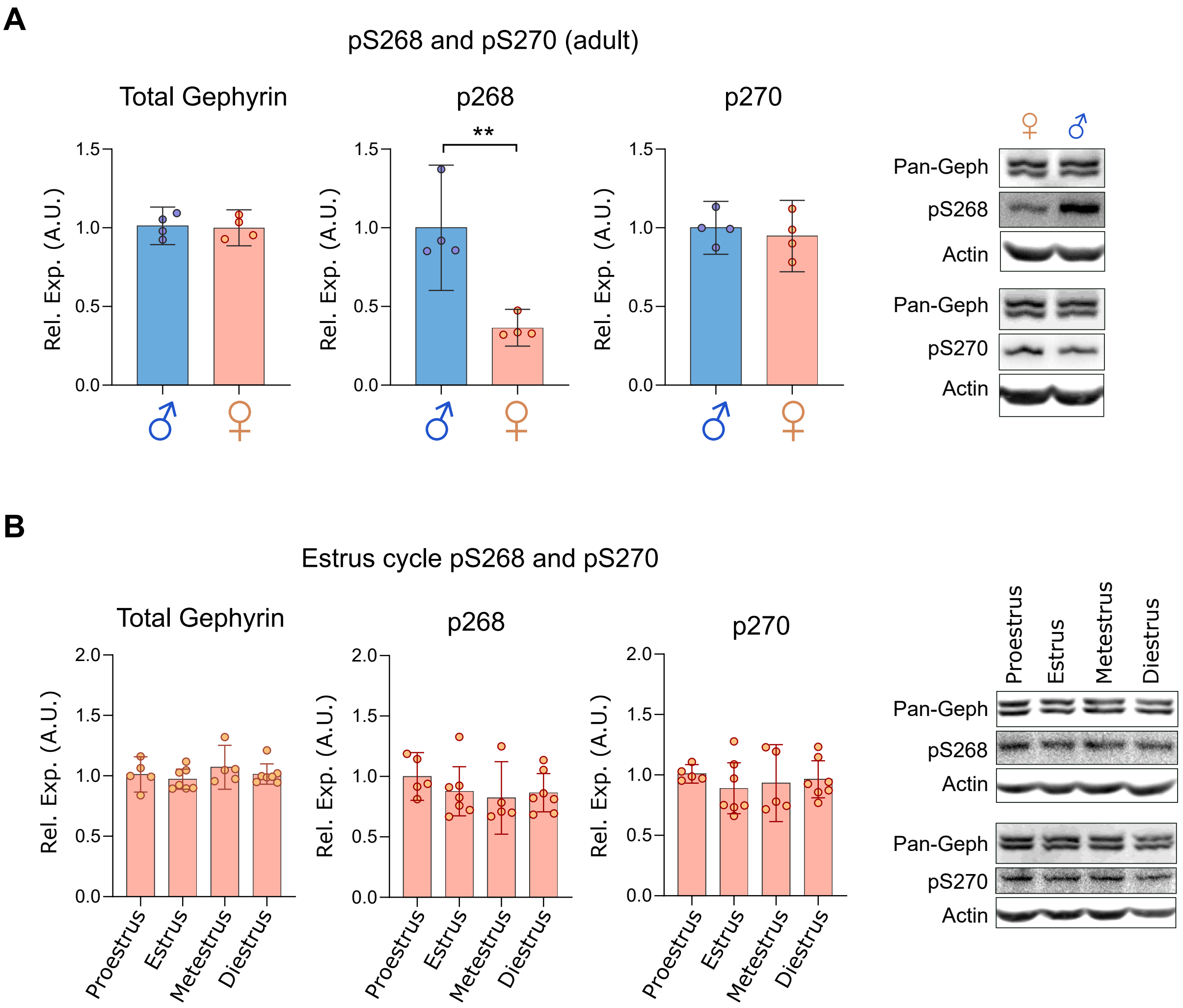

FIGURE 1 SUPPLEMENT

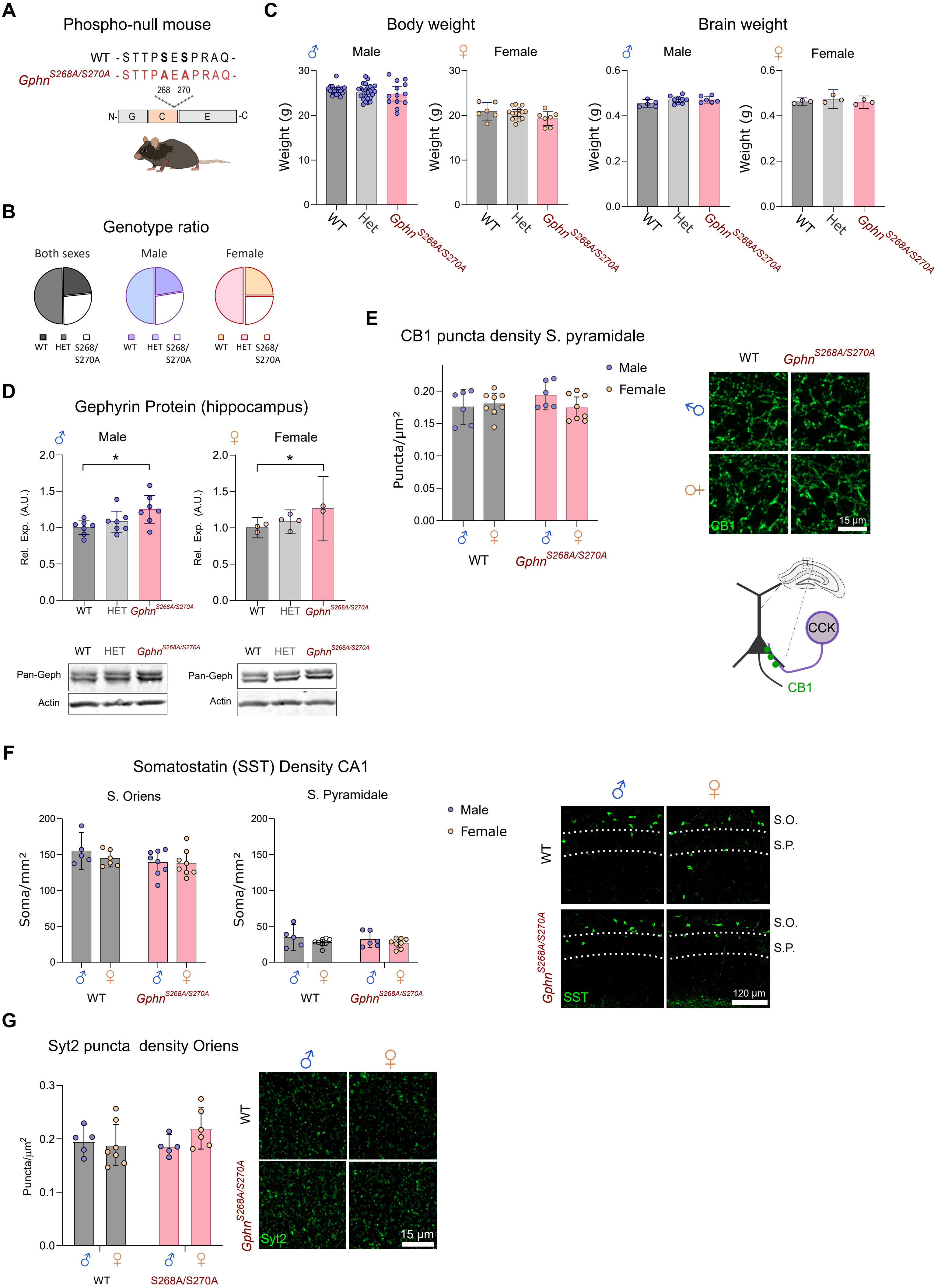

Figure 2 Supplement

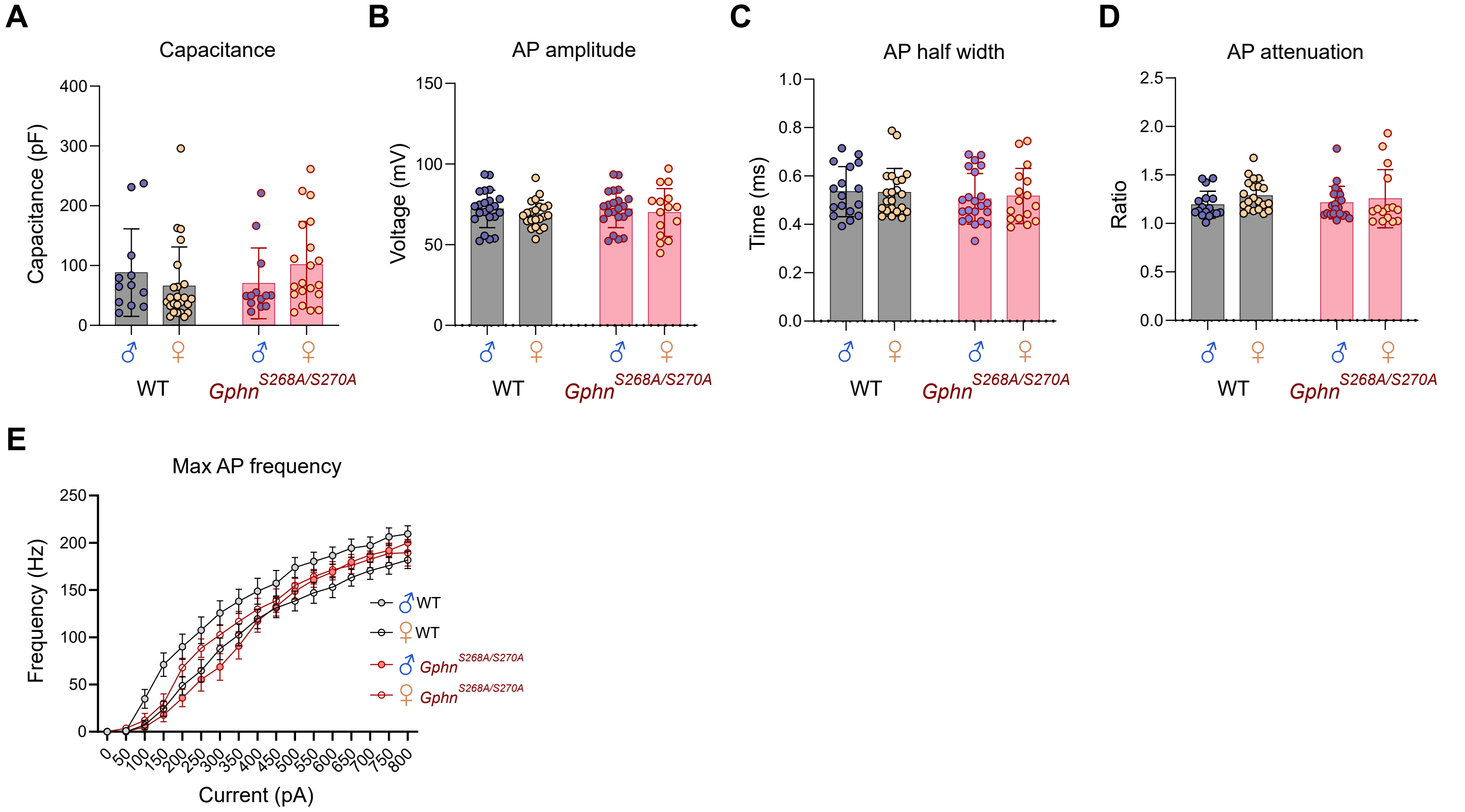

Figure 3 Supplement

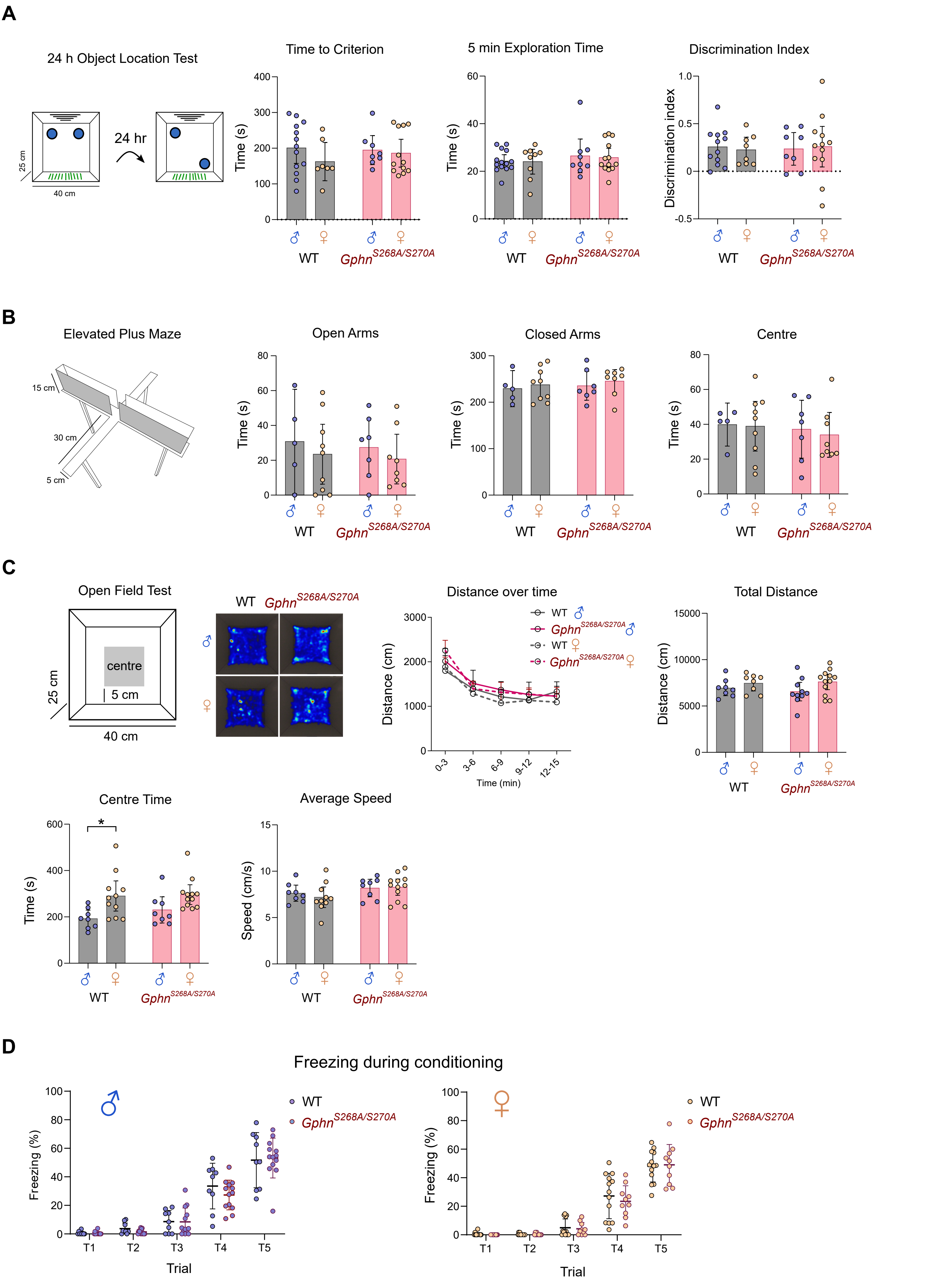

Figure 4 Supplement

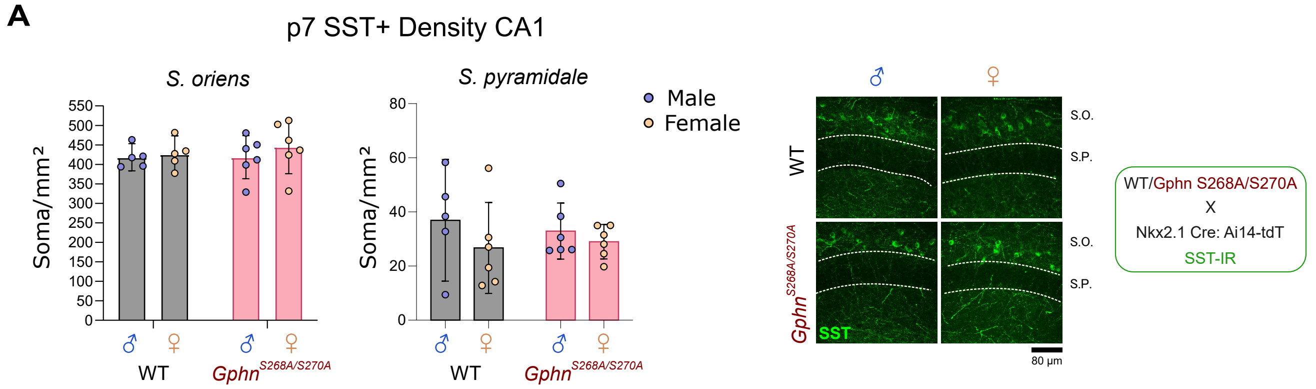

Figure 5 Supplement

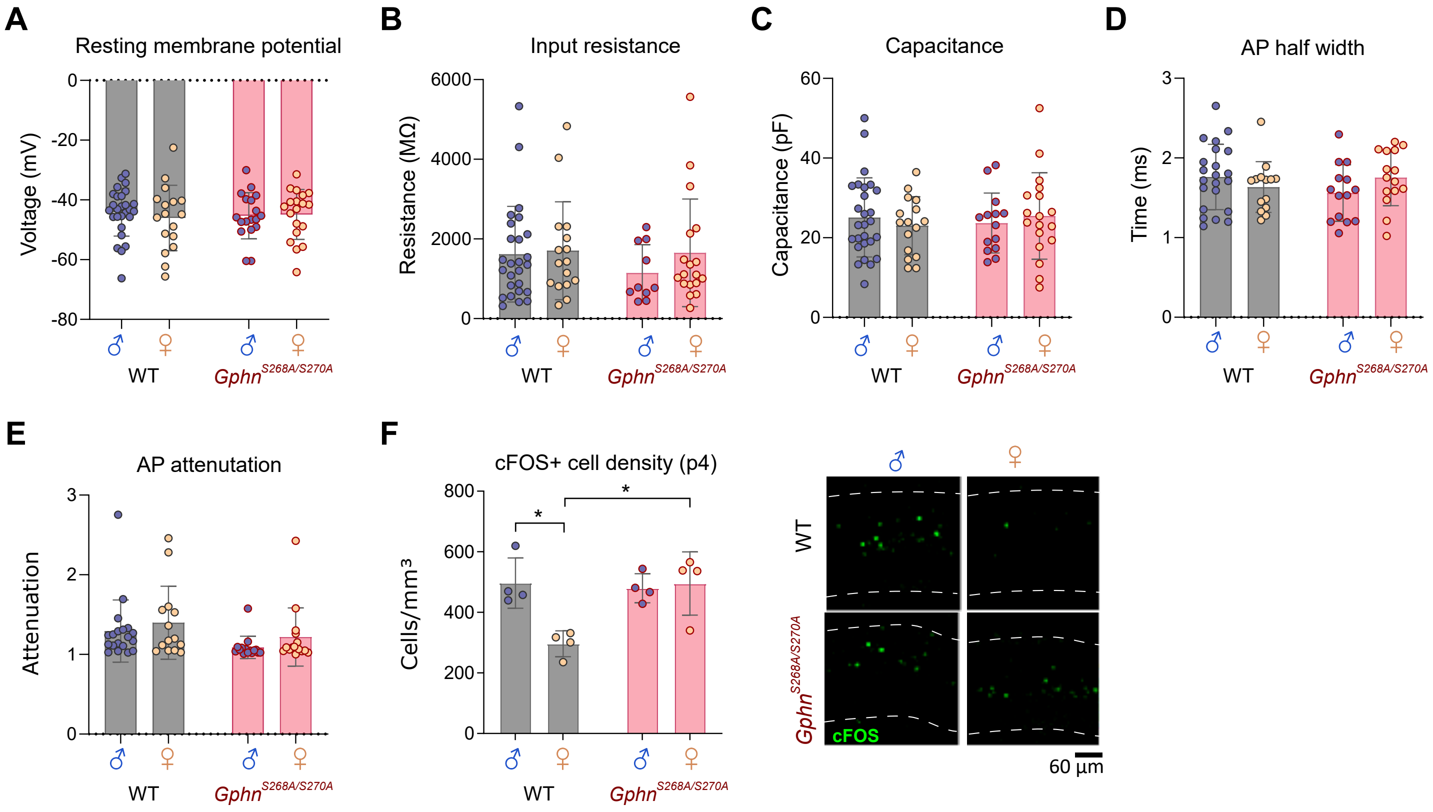

Figure 6 Supplement
